# Heterologous Biosynthesis, Modifications and Structural Characterization of Ruminococcin-A, a Lanthipeptide From the Gut Bacterium *Ruminococcus gnavus* E1, in *Escherichia coli*

**DOI:** 10.3389/fmicb.2018.01688

**Published:** 2018-07-26

**Authors:** Elvis L. Ongey, Robert T. Giessmann, Michel Fons, Juri Rappsilber, Lorenz Adrian, Peter Neubauer

**Affiliations:** ^1^Chair of Bioprocess Engineering, Institute of Biotechnology, Technische Universität Berlin, Berlin, Germany; ^2^Aix Marseille Univ, CNRS, BIP UMR 7281, Marseille, France; ^3^Institute of Biotechnology, Technische Universität Berlin, Berlin, Germany; ^4^Department of Isotope Biogeochemistry, Helmholtz Centre for Environmental Research – UFZ, Leipzig, Germany; ^5^Chair of Geobiotechnology, Institute of Biotechnology, Technische Universität Berlin, Berlin, Germany

**Keywords:** lanthipeptides, ruminococcin-A, biosynthesis, preRumA, expression, ribosomal peptide, antimicrobial peptide, mass spectrometry

## Abstract

Ruminococcin A (RumA) is a lanthipeptide with high activity against pathogenic clostridia and is naturally produced by the strict anaerobic bacterium *Ruminococcus gnavus* E1, isolated from human intestine. Cultivating *R. gnavus* E1 is challenging, limiting high-quality production, further biotechnological development and therapeutic exploitation of RumA. To supply an alternative production system, the gene encoding RumA-modifying enzyme (RumM) and the gene encoding the unmodified precursor peptide (preRumA) were amplified from the chromosome of *R. gnavus* E1 and coexpressed in *Escherichia coli*. Our results show that the ruminococcin-A lanthionine synthetase RumM catalyzed dehydration of threonine and serine residues and subsequently installed thioether bridges into the core structure of a mutant version of preRumA (preRumA^∗^). These modifications were achieved when the peptide was expressed as a fusion protein together with green fluorescence protein (GFP), demonstrating that a larger attachment to the N-terminus of the leader peptide does not obstruct *in vivo* processivity of RumM in modifying the core peptide. The leader peptide serves as a docking sequence which the modifying enzyme recognizes and interacts with, enabling its catalytic role. We further investigated RumM catalysis in conjunction with the formation of complexes observed between RumM and the chimeric GFP fusion protein. Results obtained suggested some insights into the catalytic mechanisms of class II lanthipeptide synthetases. Our data further indicated the presence of three thioether bridges, contradicting a previous report whose findings ruled out the possibility of forming a third ring in RumA. Modified preRumA^∗^ was activated *in vitro* by removing the leader peptide using trypsin and biological activity was achieved against *Bacillus subtilis* ATCC 6633. A production yield of 6 mg of pure modified preRumA^∗^ per liter of *E. coli* culture was attained and considering the size ratio of the leader-to-core segments of preRumA^∗^, this amount would generate a final yield of approximately 1–2 mg of active RumA when the leader peptide is removed. The yield of our system exceeds that attainable in the natural producer by several 1000-fold. The system developed herein supplies useful tools for product optimization and for performing *in vivo* peptide engineering to generate new analogs with superior anti-infective properties.

## Introduction

The healthcare sector of our society is currently facing a precarious situation that has been vividly described as a “new pre-antibiotic era” where it is estimated that within the next few years, most of the commonly used anti-infective agents may become ineffective due to growing increase of antibiotic resistances provoked by improper use of antibiotics in human medication and animal farming (Rios et al., 2016). Owing to their special structural, physicochemical and functional characteristics; antimicrobial peptides (AMPs) of the lanthipeptides subgroup may be employed as alternative drugs (Dischinger et al., 2014). Lanthipeptides are a group of ribosomally synthesized peptides characterized by the presence of thioether cross-linked amino acids, lanthionines/methyllanthionines, leading to a polycyclic core structure (Dischinger et al., 2009). Lanthipeptides are mostly produced by Gram-positive bacteria and more than 100 members have been described in the literature (Dischinger et al., 2014; Ongey and Neubauer, 2016).

Lanthipeptide synthetases are enzymes that catalyze posttranslational modifications (PTMs) of lanthipeptides. Those of class II lanthipeptides are dual-functional, possessing an N-terminal dehydratase domain and a C-terminal cyclase domain (Repka et al., 2017). They are generally referred to as LanM proteins (Lan is a general notation for the products of a lanthipeptide biosynthesis cluster). As a general mechanism, the dehydratase domain of LanM catalyzes the dehydration of threonine residues in the core peptide to didehydrobutyrine (Dhb), and serine residues to didehydroalanine (Dha) (Chatterjee et al., 2005). Subsequently, the cyclase domain of LanM engages in Michael-type addition-cyclisation reactions involving Dhb/Dha and thiol groups of cysteines in the core structure to generate methyllanthionine/lanthionine (MeLan/Lan) bridges (Dabard et al., 2001). Once all the PTMs are installed, the N-terminal domain of a dedicated bifunctional ABC-transporter maturation and secretory (AMS) protein cleaves off the leader peptide, consequently activating the peptide which is then exported by the same protein to the extracellular space (Repka et al., 2017).

We have previously shown that compared with chemical approaches biotechnological methods are superior for the production of lanthipeptides (Ongey and Neubauer, 2016), and examined the beneficial effects of structural engineering on lanthipeptide pharmacological properties that may encourage therapeutic use (Ongey et al., 2017). It is, however, challenging to develop a robust bioprocess with the natural isolates since most of them like *Actinoplanes spp.* (Boakes et al., 2010), *Actinomadura namibiensis* (Meindl et al., 2010) and *Ruminococcus gnavus* (Dabard et al., 2001) produce low yields and require much efforts and cost to cultivate.

A heterologous host like *Escherichia coli* has obvious advantages including short generation time, high cell density growth, high product yield, and ease of manipulation, which may facilitate a variety of investigations on a target product. However, *E. coli* hosts do not usually harbor the machinery necessary to introduce the PTMs on the lanthipeptides and those are therefore required to be supplied to them from external sources. Nagao and colleagues isolated a gene fragment from the biosynthesis cluster of the lantibiotic (lanthipeptide with antibacterial activity) nukacin ISK-1, containing the genes that encode the precursor peptide and the lanthionine synthetase, and expressed them on a single vector in *E. coli* to obtain modified nukacin ISK-1 (Nagao et al., 2005). In similar approaches several other highly efficient lanthipeptide production systems were described in recent years where the essential genes were successively inserted in a single vector under the control of separate T7-RNA polymerase promoters (Fujita et al., 2007; Caetano et al., 2011a; Shi et al., 2011, 2012; Tang and van der Donk, 2012; Kuthning et al., 2015). Recently, data obtained by Basi-Chipalu and coworkers varied the concept by coexpressing the precursor peptide and the lanthionine synthetase on separate plasmids in *E. coli* to obtain modified pseudomycoicidin (Basi-Chipalu et al., 2015), although mixtures of incomplete dehydration products were observed.

**FIGURE 1:**
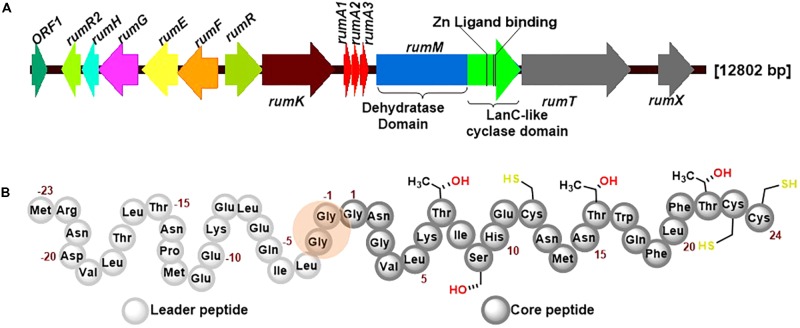
Ruminococcin-A biosynthesis genome cluster and primary structure of the precursor peptide preRumA. **(A)** The genetic cluster encodes *rumFEGHR2*, hypothesized to confer immunity in the host, *rumRK*, encoding regulatory proteins, *rumTX*, encoding proteins involved in transport and processing*, rumA1A2A3* encoding the preRumA and *rumM*, encoding the dual-functional lanthionine synthetase (RumM). The various catalytic domains of RumM, including active site cofactor of the cyclase domain are indicated. **(B)** The peptide sequence of preRumA contains a leader peptide (position –23 to –1), the core peptide (position 1–24) and the Gly-Gly motif (position –2/–1) where proteolytic cleavage occurs. Threonine, serine and cysteine side chains involved in thioether crosslink formation are also indicated.

RumA is a trypsin-regulated lantibiotic naturally produced by *R. gnavus* E1 (Dabard et al., 2001; Gomez et al., 2002). *R. gnavus* E1 is a strictly anaerobic Gram-positive bacterium which was first identified in human feces (Ramare et al., 1993). This AMP possesses high activity against pathogenic *Clostridia*, making it a plausible target for the treatment of human infections and livestock diseases, and may also be used in food industry as preservative. **Figure 1A** shows the biosynthesis genes cluster of RumA, with the three genes *rumA1A2A3* coding for the same product (preRumA) – possibly an outcome of genetic multiplication that occurred during evolution. The other essential genes include *rumM* which encodes the dual-functional lanthionine synthetase (RumM) that catalyzes PTMs in the core peptide (**Figure 1B**). *rumT* encodes the bifunctional AMS protein responsible for cleaving off the leader peptide (**Figure 1B**) and consequently activating the modified preRumA. The leader peptide serves as a docking motif which allows the modifying enzyme to bind and catalyze modifications in the core peptide, and also keeps the peptide inactive in the producer organism (Mavaro et al., 2011; Repka et al., 2017). Although Ramare et al. (1993) suggested that trypsin was the activator protein, subsequent studies proved that it is actually involved in the regulation of RumA biosynthesis (Gomez et al., 2002). Additionally, although a trypsin site is present in the core structure of RumA (Lys6), Dabard et al. (2001) showed that trypsin does not cleave at this site due to the presence of modifications at the adjacent threonine residue.

Expression and peptide engineering possibilities of RumA may be limited by the strictly anaerobic nature of the native producer and the relatively large size (12.8 kb) of the ruminococcin-A gene cluster (Gomez et al., 2002). As reported for the class II lanthipeptides nukacin ISK-1 (Nagao et al., 2005) and lichenicidin (Caetano et al., 2011b), and many other lanthipeptides (Shi et al., 2011), active expression in *E. coli* does not require all the genes present in the biosynthesis cluster. This is because most of the other genes present in the biosynthesis cluster play accessory roles and are not directly involved in the expression and PTMs formation process. Therefore, in order to limit metabolic burden on a heterologous host, the “non-essential” genes may be isolated to facilitated high-quality production and purification. For instance, the hypothetical genes for immunity *rumFEGHR2*, the bifunctional transporter *rumT* and regulatory operon *rumRK* may not directly involved in the biosynthesis and modifications of preRumA. Taking this into consideration, we posited that the biosynthesis and modification of preRumA solely requires the expression of *rumA* and *rumM* genes. In this study, we utilized dual-vector and single-vector approaches to engineer the pathway for biosynthesis and modifications of preRumA in *E. coli.* The genes encoding the lanthionine synthetase and the precursor peptide were amplified from the genome of *R. gnavus* E1 and coexpressed in *E. coli*. We show that the biosynthesis and modification of preRumA can be successfully achieved by expressing the precursor peptide as a cleavable fusion protein together with GFP. A simple extraction strategy was developed to isolate the cleaved peptide and mass spectrometry was applied to characterize the structure of the peptide. The heterologous system developed in this study may be applied in subsequent studies as a useful tool for optimizing ruminoccin-A production, as well as performing *in vivo* peptide engineering to improve its physicochemical and pharmacological features or generating new analogs of the peptide with superior anti-infective properties.

## Materials and Methods

### Bacterial Strains and Cultivation Conditions

*Ruminococcus gnavus* E1 cultivation was performed in an anaerobic cabinet at 37°C, using a pre-reduced brain heart infusion (Difco Laboratories, Detroit, MI, United States) broth into which 5 g of yeast extract (Difco) and 5 mg of hemin (Sigma-Aldrich) per liter were added as supplements (Dabard et al., 2001). *E. coli* Top10 was used for cloning and plasmid storage while *E. coli* W3110 was used as the expression host. For *E. coli* cultivations, all media were supplemented with antibiotic concentrations of 50 μg ml^-1^ of kanamycin or 34 μg ml^-1^ of chloramphenicol. Unless stated otherwise, *E. coli* and *B. subtilis ATCC 6633* cultivations were performed at 30°C with an agitation speed of 200 rpm. All strains used in this study are described in **Table 1**.

**Table 1:** Description of microbial strains used in this study.

Name of strain	Description	Purpose	Source
*E. coli* W3110	Wild type-like *E. coli:* F^−^λ^−^ *rp*/z-I *INV(rrnD, rrnE)*	Expression host	Stock center, Yale
*Ruminococcus gnavus* El	Wild type	Isolation of genomic DNA	CNRS, Marseille
*B.subtilis ATCC 6633*	–	Biological activity assay	DSM 347
*E. coli* Top 10	F^-^*mcrA Δ(mrr-hsdRMS-mcrBC) ϕ80lacZΔM15 ΔlacX74 recAl* araD139 Δ(ara leu) 7697 galU galK rpsL (StrR) endAl nupG	Plasmid storage	Invitrogen
WLEOv4	Cm^R^, W3110 carrying pLEO*rA*	Expression of His6-preRumA	This work
WLEO*rM*’	Cm^R^, W3110 carrying pLEO*rM*’	Expression of His6-RumM	This work
WLEO*rM*i	Cm^R^, W3110 carrying pLEO*rM1*	Expression of His6-RumM	This work
WLEOM*/M*	Kan^R^, Cm^R^, W3110 carrying pLEO*rA* and pLEOr*M*	Coexpression of His6-preRumA and His6-RumM on separate plasmid	This work
WLEO*gr*v4	Cm^R^, W3110 carrying pLEO*grA*	Expression of His6-GFP-TEV-preRumA	This work
WLEO*gr*v4*/M*	Kan^R^, *Cm^R^*, W3110 carrying pLEO*grA* and pLEO*M*	Coexpression of His6-GFP-TEV-preRumA and His6-RumM on separate plasmid	This work
WLEO*gr*.4*^∗^*	Cm^R^, W3110 carrying pLEO*grA^∗^*	Expression of His6-GFP-TEV-preRumA^∗^	This work
*WLEOgrA^∗^Ml*	*Cm^R^*, W3110 carrying pLEO*grA^∗^M1*	Coexpression of His6-GFP-TEV-preRumA^∗^ and His6-RumM on the same plasmid	This work
*WLEOsrA^∗^*	*Cm^R^*, W3110 carrying pLEO*srA^∗^*	Expression of His6-SUMO-preRumA^∗^	This work
*WLEOsrA^∗^M*	*Cm^R^*, W3110 carrying pLEO*srA^∗^M*	Coexpression of His6-SUMO-preRumA^∗^ and His6-RumM on the same plasmid	This work


### PCR Cloning, Mutagenesis, and Construction of Expression Vectors

For PCR, genomic DNA from *R. gnavus* E1 was extracted using GenElute^TM^ Bacterial Genomic DNA Kit (Sigma-Aldrich). Phusion^®^ and Q5^®^ High-Fidelity DNA polymerases were purchased from New England Biolabs (NEB) (Frankfurt am Main, Germany). Oligonucleotides (Supplementary Table S1) were purchased from TIB MOLBIOL (Berlin, Germany). For isolation and storage purposes, the primer pair *rumClus_f/rumClus_r* was used to amplify a segment of the ruminococcin-A gene cluster (GenBank accession no. AF320327), containing *rumA1A2A3* and *rumM*. The blunt-ended amplicons were directly inserted into a *SmaI*-restricted pCTUT7 vector to yield pLEO*rC2* (Supplementary Figure S1). The pCTUT7 vector belongs to a library of 45 plasmids derived from pKA100 (Krebber et al., 1996, 1997) and designed for optimizing recombinant protein expression in *E. coli* (Kraft et al., 2007). The main features of these vectors are; a *lacUV5*-based promoter system (designated as *CTU*), possessing a mutation in the catabolite activator protein (CAP) site, *lacUV5* sequence of the original pAK100, -35 region from tac promoter, ribosomal binding site (RBS) of gene 10 of bacteriophage T7 (T7 RBS), the *lac* repressor gene and a chloramphenicol resistance gene (Kraft et al., 2007). Specific plasmids from this library were used recently to obtain high yield of the non-ribosomal peptide valinomycin in *E. coli* W3110 (Jaitzig et al., 2013; Li et al., 2014), showing that the system is efficient for the production of difficult-to-express proteins.

For the expression of the precursor peptide preRumA, PCR was performed with primer pair *rumA_f/rumA_r* and pLEO*rC2* as template. The resulting *rumA1* PCR product was digested with *NheI* and *PstI* FastDigest enzymes (Thermo Scientific, Darmstadt, Germany) and ligated into *NheI*/*PstI*-digested pCTUT7 vector yielding pLEO*rA*. For the expression of ruminococcin-A lanthionine synthetase RumM, PCRs with primer pairs *rumM_f2/rumM_r* and *rumM_f3/rumM_r* using pLEO*rC2* as template were performed, and the resulting amplicons were digested with *NheI*/*PstI* and *BamHI*/*PstI*, and further ligated into the pCTUT7 and pJL10 expression vectors generating pLEO*rM*’ and pLEO*rM*, respectively. The pJL10 plasmid is a modification of commercial plasmid pRSF-1b where the T7 promoter system was replaced with the *CU* promoter (Li et al., 2015). The promoter designated *CU* is also a *lacUV5*-based and was obtained from the library created by Kraft et al. (2007). Additional features on the plasmid include a kanamycin resistance gene, RSF 1030 origin of replication and the *lac* repressor gene.

To replace the RBS sequence (TAACGAGGGCAACAT) of the promoter/RBS region of pJL10 plasmid hosting the *CU* promoter, with that of the T7 RBS (GAAGGAGATATACAT), the restriction enzymes *SpeI* and *NdeI* were used to remove the *lacI* gene and the *CTU* promoter from the pCTUT7 vector. Primer pair *SDT7_f/SDT7_r* was used to amplify the *lacI* gene together with the *CU* promoter from the pJL10 vector. The resulting amplicons were digested with *SpeI* and *NdeI* and subsequently cloned into the backbone of the previously digested pCTUT7 to yield pCUT7. *NheI*/*PstI*-digested *rumM* amplicons were inserted into *NheI*/*PstI*-restricted pCUT7 vector to yield pLEO*rM1.*

PCR, using pLEO*rA* as template together with the Q5^®^ Site-Directed Mutagenesis Kit (NEB) and the *pTrypsin_f/pTrypsin_r* primer pair were employed to replace the Gly residue at position -1 of preRumA with Arg to yield the pLEO*rA^∗^* plasmid.

To fuse preRumA or preRumA*^∗^* to green fluorescence protein (GFP), two special primers *rumA^s^_f* and *gfp^s^*_*r* were designed such that their 5^′^ overhangs had partially complementary regions. *gfpmut-2* was amplified from pUA66 plasmid (Zaslaver et al., 2004) using primer pair *gfp*_*f*/*gfp^s^*_*r* while *rumA* or *rumA^∗^* was amplified from pLEO*rA* or pLEO*rA^∗^* using primer pair *rumA^s^_f*/*rumA_r*. A TEV protease cleavage site (GAAAACCTGTATTTTCAGGGC) was also inserted in between *gfpmut-2* and *rumA*. The final fusion fragment obtained via overlap extension PCR (Horton et al., 1990) was digested with *NheI*/*PstI* and ligated into corresponding *NheI*/*PstI*-digested pCTUT7 to yield pLEO*grA* or pLEO*grA^∗^.* To fuse preRumA^∗^ to small ubiquitin-like modifier (SUMO), *NheI*/*PstI*-digested *rumA^∗^* PCR product amplified from pLEO*rA^∗^* was inserted into *NheI*/*PstI*-digested pCTUT7-SUMO plasmid to yield pLEO*srA^∗^*.

To clone the genes encoding the precursor peptide and the modifying enzyme successively on the same plasmid, primer pair *prumM_f/prumM_r* was used to amplify a cassette from pLEO*rM1* containing the *CU* promoter, T7 RBS and *rumM.* The resulting amplicons were digested with *PstI and HindIII* and subsequently inserted into *PstI/HindIII*-digested pLEO*grA^∗^* to generate pLEO*grA^∗^M1*. In the same manner, *rumM* was amplified from pLEO*rM* and inserted into pLEO*srA^∗^* to yield pLEO*srA^∗^M*. The sequences of all resultant plasmids and mutants were verified by sequencing.

### Transformation, Growth and Protein Expression

To achieve expression of the designated genes, pLEO*rA* and pLEO*grA* were cotransformed with pLEO*rM* in electrocompetent *E. coli* W3110 cells resulting in expression strain WLEO*rA/M* and WLEO*grA/M*, respectively. In a similar manner, pLEO*grA^∗^M1* and pLEOs*rA^∗^M* were transformed to yield the strains WLEO*grA^∗^M1* and WLEOs*rA^∗^M*, respectively. Additionally, pLEO*rA*, pLEO*grA*, pLEO*grA^∗^*, pLEO*srA^∗^*, pLEO*rM*’, and pLEO*rM1* were separately transformed into *E. coli* W3110 yielding WLEO*rA*, WLEO*grA*, WLEO*grA^∗^*, WLEO*srA^∗^*, WLEO*rM*’, and WLEO*rM1* strains, respectively (**Table 1**).

Growth optimization and expression tests were performed in 24 deep-well plates using the EnPresso^®^ B growth system (Enpresso GmbH, Berlin, Germany) according to the manufacturer’s recommended procedure. At the point of induction, 3 ml of respective cultures were split into the 24-well deep-well plates and induced with varying concentrations of IPTG inducer. Cells were cultivated for further 18–24 h while measuring the optical densities at different time points. At the end of cultivation, cells were collected in 2 ml Eppendorf tubes by centrifuging for 1 min at 16,000 × *g* at 4°C. Larger cultures were cultivated in freshly prepared terrific broth (TB) medium by supplying appropriate concentrations of required antibiotics to 1 l of culture. The cells were induced with 100 μM IPTG (optimized from micro-scale cultivations) at OD_600_ between 0.8 and 1, and further cultivated for 18–24 h. Cells were collected by centrifugation for 10 min at 6000 × *g* at 4°C. The cell pellets were stored at -20°C for further preparations and analysis.

### Purification, TEV Cleavage and Extraction

Cell pellets from -20°C were thawed and resuspended in lysis buffer (50 mM NaH_2_PO_4_⋅H_2_O, pH 8, 300 mM, 10 mM Imidazole). Sonication was applied to disrupt cells and release proteins. Cell debris was separated from the soluble lysate by centrifugation for 15 min at 16,000 × *g* at 4°C. The clarified lysates were applied to His SpinTrap columns (GE Healthcare, United Kingdom) for expression/purification screening. Larger purifications were performed with the ÄKTA Avant 25 instrument by loading the clarified bacterial lysates onto a 1-ml HisTrap FF Crude column (all from GE Healthcare) and eluted by applying a gradient range of 0–100% of elution buffer (50 mM NaH_2_PO_4_⋅H_2_O pH 8, 300 mM NaCl, 500 mM imidazole). Purified His6-RumM was concentrated using Amicon Ultra centrifugation tubes with 100 kDa molecular weight cut-off (Merk Millipore, Darmstadt) while His6-GFP-TEV-RumA^∗^ was concentrated using a 30-kDa cut-off tube. Protein concentrations were measured using the Bradford Coomassie brilliant blue dye method (Bradford, 1976). The IMAC concentrated sample was subsequently injected onto a HiLoad 16/60 Superdex pg 200 column (GE Healthcare) and eluted with the gel filtration buffer (20 mM NaH_2_PO_4_⋅H_2_O pH 8, 150 mM NaCl, 10% glycerol). Fractions were pooled, concentrated and measured using the Bradford assay, aliquoted and stored at -20°C.

Before TEV cleavage the samples from -20°C were thawed on ice, and 1 mM DTT and 0.5 mM EDTA were added. One Hundred microgram of fresh homemade TEV protease was added to 1 mg of protein and then incubated overnight at 4°C. TEV-digested samples were further purified using a Ni^2+^-NTA column and collecting the eluent containing the desired product. The eluent was then dialyzed in water and dried using a Concentrator Plus vacuum (Eppendorf, Hamburg, Germany) and stored at -20°C for further use. Alternatively, the TEV-digested samples were extracted with 1-butanol by adding one volume of the solvent and stirring the mixture at room temperature for 1–2 h. Extracts were separated by centrifugation for 5 min at 3000 × *g*. The upper organic layer containing the peptides was obtained and protein concentrations were measured using the NanoDrop. Recovered peptide extracts were dried and stored at -20°C for subsequent applications.

### SDS-PAGE and Native PAGE Analyses

For sodium dodecyl sulfate polyacrylamide gel electrophoresis (SDS-PAGE), larger proteins were processed with 10% gels using standard protocols (Sambrook and Russell, 2001) while smaller peptides were analyzed using 16% Tricine PAGE as described elsewhere (Schägger, 2006). For native PAGE, samples, buffers and gel recipes were assembled following the same procedure as described for standard SDS-PAGE except that detergents (SDS and urea), DTT and sample heating step (95°C, 5 min) were not included. Eight percent resolving, with 4% stacking gels were used for the native PAGE analysis. All gels were visualized by staining with colloidal blue silver Coomassie G-250 as reported elsewhere (Candiano et al., 2004).

### Automated Fluorescence Measurements

Qualitatively, target product expressions in WLEO*grA^∗^* and WLEO*grA^∗^M1* were estimated by measuring the GFP fluorescence signal intensities. The strains were cultivated in 24-well round-bottom plates at 30°C, and the relative fluorescence emission of the cells was measured using a Synergy^TM^ Mx monochromator-based multimode microplate reader (BioTek Instruments, Winooski, Vermont, United States). Time-course GFP fluorescence signal intensities were measured and recorded as relative fluorescent unit (RFU) every 10 min at 528 nm for 15–30 h. As a control the auto-fluorescence of a strain which does not express GFP was also recorded in the same manner.

### Mass-Spectrometric Analyses of preRumA^∗^

For peptide identification, pieces were excised from SDS-PAGE gels and digested with an in-gel tryptic digestion protocol (Shevchenko et al., 2006). Dried samples were redissolved in 0.1% formic acid. Both the tryptic digest and resolubilized samples were desalted and concentrated using C_18_-ZipTips (Merck, Darmstadt, Germany). When desired, the ZipTip procedure was also applied directly to the purified TEV-digested samples. Processed samples were directly injected into an LTQ Orbitrap XL Hybrid Ion Trap-Orbitrap mass spectrometer or nanoLC-coupled Thermo Orbitrap Fusion mass spectrometer (Thermo Fisher Scientific), both with a nanoelectrospray ion source. For the LTQ-Orbitrap, highly intense ions identified in the full MS were directed to the orbitrap analyzer where they were fragmented and processed in the linear ion trap. For the Thermo Orbitrap Fusion, specific ions were selected and channeled to the ion-routing multipole where they were fragmented, processed in the C-trap and analyzed by the orbitrap mass analyzer.

For the liquid chromatography-electrospray ionization-mass spectrometry (LC-ESI-MS) analyses, the dried extracts were dissolved in acetonitrile (ACN)/H_2_O/formic acid (50:50:0.2%). Five microliter were introduced into an Agilent 1290 Infinity HPLC system (Agilent Technologies, Waldbronn, Germany), followed by an ESI-Triple-Quadrupole LC-MS 6460 electron spray ionization mass spectrometry analysis using multiple reaction monitoring. The material used for the column and pre-column was Poroshell 120 EC-C8 (2.1 × 50 mm, 2.1 μm). Solvent A of the HPLC system corresponded to H_2_O while ACN was designated as solvent B. The introduced sample was eluted with an ACN gradient from 5 to 20% solvent B within 0.5 min, followed by 70% B in 4 min and then to 100% B in 0.2 min. An isocratic elution with 100% B was finally applied for 1.3 min. The flow rate was set at 0.7 ml min^-1^. Xcalibur (Thermo Fisher Scientific), MaxQuant (Cox and Mann, 2008) and Xi Spectrum Viewer^1^ were used for data analysis. MASCOT search engine (Matrix Science, United Kingdom) was used for sequence identification by searching the MS and MS/MS data.

### Iodoacetamide Derivatization and Trypsin Digestion

Dried extracts were dissolved in 100 mM ammonium bicarbonate to obtain ≈0.5–1 mg ml^-1^ of soluble peptide. DTT was added to 30 μl of the soluble peptide to a final concentration of 10 mM. The mixture was incubated at 55°C for 1 h. Four hundred millimolar Iodoacetamide (Sigma) (freshly dissolved in 100 mM ammonium bicarbonate) was added to the sample to a final concentration of 25 mM and incubated at room temperature for 45 min in the dark. Six point three microliter of 0.1 μg μl^-1^ mass spectrometry grade trypsin (Promega, Lyon, France) solution was added to the derivatized sample and incubated on a shaking platform at 37°C overnight. The samples were purified using C_18_-ZipTip and analyzed as described earlier. For the modified and non-modified His6-SUMO-preRumA^∗^, the derivatization step was not performed. The purified samples in elution buffer 2 (20 mM TRIS-HCl, pH 8, 250 mM NaCl, 300 mM imidazole) were first dialyzed in water and further buffer-exchanged in 100 mM ammonium bicarbonate buffer. Fifteen microliter of trypsin stock solution (0.1 μg μl^-1^) was added to 100 μl of samples obtained from each of the modified and non-modified His6-SUMO-preRumA^∗^ and incubated on a shaking platform at 37°C overnight. The whole tryptic digests were used for SDS-PAGE and bioassay analyses.

### Biological Assay

*Bacillus subtilis ATCC 6633* is one of the indicator strains for RumA as reported by Dabard et al. (2001). The strain was cultivated in a medium composed of peptone (5.0 g l^-1^), meat extract (3.0 g l^-1^) and MnSO_4_ (0.02 g l^-1^) and then spread on bacteriological agar plates (1.5% agar) containing punched wells. The plates were kept under the clean bench for about 15 min to allow excess media to evaporate and 50 μl of each digested sample were pipetted into separate wells and incubated at 30°C overnight.

## Results

### Construction of Vectors for *E. coli* Expression of preRumA

The genes *rumA* and *rumM* were transferred to an *E. coli* host on individual plasmids with compatible origins of replication. *rumM* was encoded in three different plasmid versions pLEO*rM*, pLEO*M1*, and pLEO*M1* together with a His-tag (His6-RumM). *rumA* was also encoded in three different plasmid versions pLEO*rA* (encoding His6-preRumA), pLEOsrA^∗^ (encoding His6-SUMO-preRumA^∗^) in which SUMO was fused to the N-terminus of preRumA^∗^, pLEO*grA* (encoding His6-GFP-TEV-preRumA) and pLEO*grA^∗^* (encoding His6-GFP-TEV-preRumA^∗^). The latter two constructs contained GFP which was linked to the precursor peptide via a TEV cleavage site. Note here that the symbol (^∗^) denotes the variant containing trypsin cleavage site at position -1 of preRumA which was generated by replacing Gly-1 with Arg. For clarity, this mutant is referred to as preRumA^∗^ while the wild-type remains preRumA throughout this report. Additionally, plasmid versions encoding a bicistronic operon pLEO*grA^∗^M1* (expressing both His6-RumM and His6-GFP-TEV-preRumA^∗^) and pLEO*srA^∗^M* (expressing both His6-RumM and His6-SUMO-preRumA^∗^) were also generated. The plasmids containing the two *rumA* variants and *rumM* were traceable via different antibiotic resistance genes.

Our cloning approach employed the use of conventional restriction enzymes and ligation to insert the specific genes of interest under the control of IPTG-inducible promoters. Two main promoter systems were employed: the IPTG-inducible *CTU* and *CU* promoters which are based on the *lacUV5* promoter system (Kraft et al., 2007). The vector that hosted the *CTU* promoter was based on pKA100 (Krebber et al., 1996) and pDest15 and possessed mutation in the CAP site, *lacUV5* sequence of the original pAK100, -35 region from *tac* promoter, T7 RBS and a recombination cassette amplified from pDest15 (Kraft et al., 2007). Additionally, the plasmid that hosted the *CU* promoter (pJL10) was a modification of pRSF-1b where the strong T7 promoter was substituted for the *CU* promoter (Li et al., 2015). The difference between the *CTU* (stronger) and *CU* (relatively weaker) promoters were specific mutations at their respective -35 regions and RBS (Kraft et al., 2007). The plasmid maps of the resultant expression vectors used in this study are described in (Supplementary Figure S2).

### Expression of His6-preRumA and His6-GFP-TEV-preRumA(^∗^) Constructs

**FIGURE 2:**
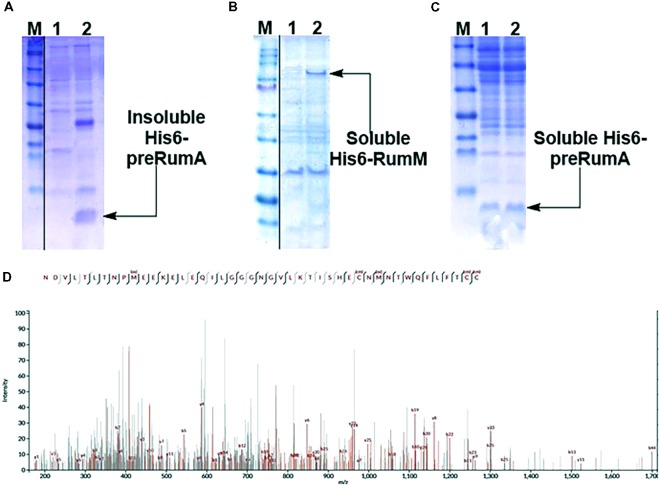
Expression test and MS^2^-ESI-Orbitrap spectrum of gel-extracted His6-preRumA. **(A)** SDS-PAGE analysis of purified soluble (lane 1) and insoluble (lane 2) His6-preRumA produced in the absence of RumM (i.e., extracts from strain WLEO*rA*). **(B)** Analysis of purified control extracts from strain WLEO*rA* (Lane 1) and extracts from strain WLEO*rA/M*, showing soluble production of His6-RumM (Lane 2). **(C)** Soluble His6-preRumA extracted from strain WLEO*rA/M* (Lanes 1 and 2). **(D)** Complete fragmentation of His6-preRumA, following tryptic digestion. None of the precursor ions resulting from the LC-MS analysis of the tryptic digest indicated fragmentation restrictions at the positions where thioether cross-bridges were expected to be formed. Consequently, there was no ring structure in the product measured. cm, carbamidomethylation; ox, oxidation. Full scans of sliced gel pieces in A, B and C are presented in Supplementary Figure S9.

Expressing His6-preRumA alone (i.e., applying the strain WLEO*rA*) did not produce any soluble product. His6-preRumA was largely present in the insoluble fraction (**Figure 2A**), suggesting that the his-tagged precursor peptide must have aggregated or formed inclusion bodies as reported for other lantipeptide precursors expressed in the absence of the PTMs enzyme (Li et al., 2009, 2010). Nevertheless, when His6-preRumA was coexpressed with His6-RumM, SDS-PAGE analyses of extract from the strain WLEO*rA/M* indicated soluble expression of both His6-RumM (**Figure 2B**) and His6-preRumA (**Figure 2C**). Excised SDS-PAGE bands representing His6-preRumA and His6-RumM were subjected to in-gel tryptic digestion and analyzed using an Orbitrap-coupled HPLC system. Subsequent MASCOT search of the MS and MS^2^ data from orbitrap analyzer identified the preRumA peptide sequence (UniProt ID: P83674) as well as RumM sequence (UniProt ID: Q9L3F1). No modification was identified in His6-preRumA extracted from strain WLEO*rA/M*. Furthermore, MS^2^ data of tryptic digest of the His6-preRumA did not provide any supporting evidence to suggest that the precursor peptide was modified in the two-vector system (**Figure 2D**), even though expression of RumM was visibly apparent.

Since we did not achieve modifications of preRumA by coexpressing His6-preRumA simultaneously with RumM, and considering the fact that the small precursor peptide once expressed may encounter diverse physical and biochemical challenges prior to PTMs formation by RumM, we decided to provide a physical support by fusing GFP to its N-terminus via a TEV cleavage site as described in the experimental part. This was done to enable solubility and stability of the precursor peptide. Expressing the resulting chimeric construct encoding the His6-GFP-TEV-preRumA alone or coexpressing in the presence of low levels of RumM, encountered some degradation issues (Supplementary Figure S3) as described in Supplementary Information section “Fusion of preRumA to GFP, Expression and LC-ESI-MS Analyses.” A mixture of partially modified, fully modified and non-modified preRumA were identified in butanol extracts of TEV-digested His6-GFP-TEV-preRumA from strain WLEO*grAM*. The latter outcome led us to posit that the preRumA/RumM ratio was not optimal for full modifications to occur. Hence, we optimized the expression constructs and the host vectors to enhance soluble overexpression of preRumA and RumM as an approach to limit the degradation and improve production quality and quantity.

Initially, we substituted Gly-1 in preRumA for arginine to enable trypsin cleavage at that position and consequent activation of the peptide. Fusing this variant to GFP resulted in a construct encoding His6-GFP-TEV-preRumA^∗^. Furthermore, the chimeric construct together with the gene encoding His6-RumM were hosted in the same operon, generating a single-plasmid bicistronic expression vector named pLEO*grA^∗^M1*. In the bicistronic operon, expression of His6-GFP-TEV-preRumA^∗^ was controlled by the *CTU* promoter and T7 RBS. Expression of His6-RumM was enhanced by changing the RBS from the lactose operon (that was originally used together with the *CU* promoter) to T7 RBS. This implies that RumM expression was regulated by the combination of the *CU* promoter and the RBS of gene 10 of bacteriophage T7.

**FIGURE 3:**
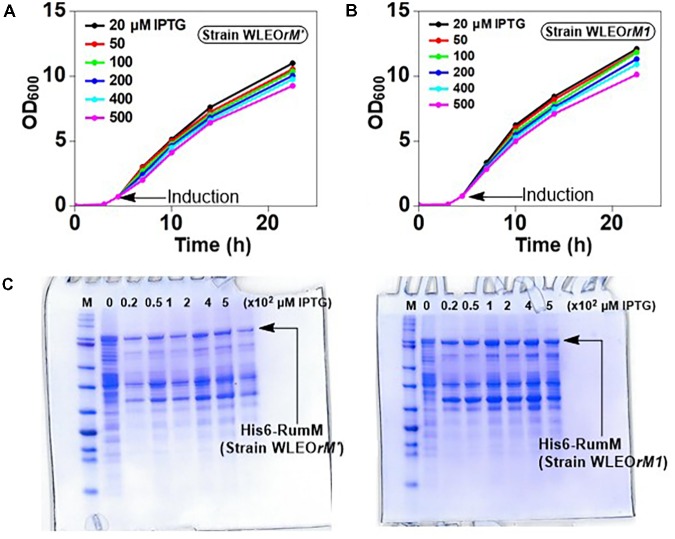
Growth and product optimization. Growth curves of strain WLEO*rM1* **(A)** and strain WLEO*rM*’ **(B)** cultures induced with varying IPTG concentrations in TB. **(C)** Expression of His6-RumM in pCTUT7 (strain WLEO*rM*’) versus pCUT7 (strain WLEO*rM1*). The concentrations of IPTG are indicated in the plots and at the top of each lane on the gel. The optimal IPTG concentration range is 50–100 μM.

To assess the strength of the different promoter arrangements, strains WLEO*rM1* (producing His6-RumM under control of *CU* promoter) and WLEO*rM*’ (producing His6-RumM under control of *CTU* promoter) were grown in TB medium as described in the experimental part except for the fact that the 100-ml culture was distributed into 24-well deep-well plate at the point of induction. Varying IPTG concentrations were applied to one row of the wells and then replicated for the remaining three rows. ODs of the cultures were measured at different time points. Results in **Figures 3A,B** show that both strains shared similar growth characteristics. His6-RumM production was drastically improved in strain WLEO*rM1* compared to strain WLEO*rM*’ (**Figure 3C**). Since there was no significant influence on growth for both plasmids, we can only conclude that the *CU* promoter and T7 RBS combination favored expression of His6-RumM. It is important to also state that although the combination of *CTU* promoter and T7 RBS is considered the strongest in that series (Kraft et al., 2007), His6-RumM expression was still low (i.e., applying pLEO*rM*’ expression vector). However, combining the *CU* promoter and T7 RBS (i.e., applying pLEO*rM1* expression vector) intensified RumM expression. Furthermore, the truncation issues encountered when His6-GFP-TEV-preRumA was expressed without or with low levels of RumM was resolved by applying the pLEO*grA^∗^M1* expression vector as seen below.

**FIGURE 4:**
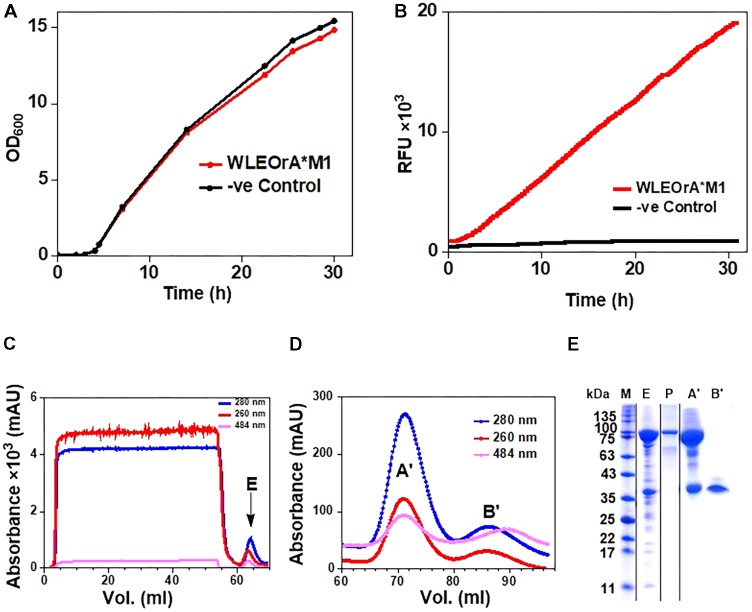
Expression and purification of His6-GFP-TEV-preRumA fusion constructs. **(A)** Growth profile of WLEO*grA^∗^M1* in TB medium. **(B)** GFP fluorescence signals of the WLEO*grA^∗^M1* strain together with that of WLEO*rM*’ (expressing His6-RumM only). **(C)** Chromatogram showing IMAC purification of extract from WLEO*grA^∗^M1.* **(D)** Size exclusion chromatograms showing elution of injected IMAC Ni^2+^-NTA sample. The absorption peak of GFP is observed at 484 nm. **(E)** SDS-PAGE analyses of the elution peaks. The letter atop gel represent; P, purified His6-RumM; E, A^′^ and B^′^ are pooled fractions obtained from respective peaks labeled in **(C,D)**.

The *E. coli* W3110 strain transformed with the pLEO*grA^∗^M1* vector named strain WLEO*grA^∗^M1* was cultivated in TB medium induced with 200 μM IPTG. The cells grew exponentially to a final OD_600_ of ∼15 after 32 h of cultivation (**Figure 4A**). The GFP fluorescence signals of WLEO*grA^∗^M1* increased constantly over the entire cultivation period while the control strain WLEO*rM*’ (expressing His6-RumM alone) remained relatively constant (**Figure 4B**). Desired proteins from strain WLEO*grA^∗^M1* lysate were purified using IMAC, followed by size exclusion chromatography. Äkta chromatograms depicting the IMAC and size exclusion chromatographic elutions of His6-RumM and His6-GFP-TEV-preRumA^∗^ are shown in **Figures 4C,D**, respectively. Purification yields are presented in **Table 2**. It was surprising to observe that from the optimized strain, His6-RumM and His6-GFP-TEV-preRumA^∗^ despite their size difference (∼70 kDa) coeluted together and appeared as a single peak on the size exclusion chromatogram (peak A^′^, **Figure 4D**), which was further confirmed by SDS-PAGE (**Figure 4E**, lane A^′^). Additionally, individual fractions that constituted the chromatographic peak A^′^ were analyzed via SDS-PAGE and results also supported this finding (Supplementary Figure S5A). This observation was further investigated by native PAGE (described later). Moreover, pooled fractions from elution peaks B^′^ produced a single band on SDS-PAGE corresponding to the molecular sizes of His6-GFP-TEV-preRumA^∗^ (**Figure 4E**, lane B^′^).

**Table 2:** Purification yields of total protein per liter.

Purification step	His6-GFP-TEV-preRumA (mg)	His6-GFP-TEV-preRumA^∗^ (mg)	^¤^His6-GFP-TEV-preRumA^∗^ (mg)	mpreRumA (mg)	^∗^(mg)	^¤^preRumA^∗^ (mg)
IMAC	84.53	97.61	97.61	–	–	–
Size exclusion	65.32	39.01	79.40	–	–	–
TEV-digested IMAC FT	–	–	–	3.23	4.45	6.14
TEV-digested butanol extract	–	–	–	1.34	2.50	4.75


*The symbol ¤, pooled fractions from peaks A^′^ and B^′^ of **Figure 4D**. This specifically applies to His6-GFP-TEV-preRumA^∗^ which appeared in two separate chromatographic peaks during purification. FT, flow-through. Concentrations were determined using the Bradford and the NanoDrop techniques.*

### *E. coli* Extraction and nLC-ESI-MS of preRumA^∗^

**FIGURE 5:**
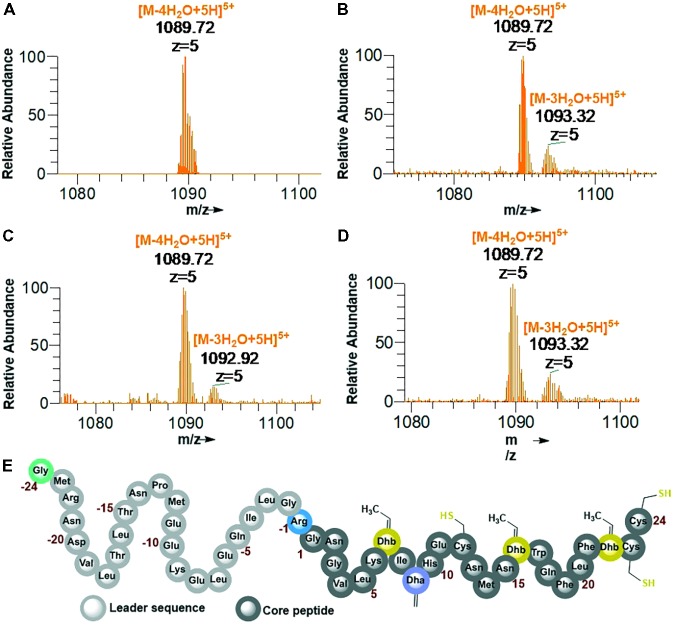
Extraction of preRumA^∗^ from TEV-digested His6-GFP-TEV-preRumA^∗^ produced in *E. coli* and nLC-ESI-MS analyses. **(A)** EIS-MS spectrum of butanol extract of TEV-digested His6-GFP-TEV-RumA^∗^. **(B)** EIS-MS spectrum of flow-through obtained from TEV-digested His6-GFP-TEV-Rum^∗^A followed by IMAC Ni^2+^-NTA column run. **(C)** Aqueous phase of butanol extract. **(D)** Direct C_18_-ZipTip purification of crude His6-GFP-TEV-preRumA^∗^ TEV digest. **(E)** Schematic structure of fully dehydrated core peptide of preRumA^∗^. Gly-24 is added to the N-terminus of the peptide after TEV cleavage. Gly-1 in the wild-type preRumA was substituted for Arg to enable trypsin cleavage. (*M*+5H)^5+^ = 1104.12.

His6-GFP-TEV-preRumA^∗^ construct purified from WLEO*grA^∗^M1* was digested with TEV protease (see Supplementary Figure S5B for SDS-PAGE analysis). One half of the digested product was extracted with butanol; the other half was run through a Ni^2+^-NTA column to remove the His6-GFP-TEV fragment and other accompanying impurities. The flow-through was collected and dialyzed in water. Both samples were dried using a vacuum concentrator. The mass spectrum of the butanol extract obtained from an Orbitrap Fusion nLC-ESI-MS analysis indicated a fourfold dehydrated product (**Figure 5A**) while the mass spectrum of IMAC flow-through extracts (processed and analyzed in the same manner) showed a mixture of fourfold dehydrated preRumA^∗^ and traces of threefold dehydrated version of the same peptide (**Figure 5B**). The measured m/z of preRumA^∗^ was consistent with the calculated m/z value of 1089.72 for the quintuple-charged ion (M-4H_2_O+5H)^5+^ resulting from a fourfold dehydrated preRumA^∗^. The mass accuracies for all MS measurements expressed in parts per million (ppm) are recorded in (Supplementary Table S2). The results here suggest that the dehydratase domain of the coexpressed RumM successfully catalyzed modifications of preRumA^∗^, associated with a mass loss of four water molecules. Comparison with the literature suggests, that these dehydrations could have occurred at Thr7, Thr16, and Thr22 to yield three Dhb, and Ser9 to produce one Dha. These results in corroboration with those discussed in Supplementary Information section “Purification and TEV Cleavage of preRumA^∗^,” supplied evidence that *in vivo* biosynthesis and modification of preRumA^∗^ is achievable in *E. coli*.

We observed that butanol extraction isolated only the fourfold dehydrated product as traces of triple-dehydrated preRumA^∗^ could be found in the flow-through extract but not in the butanol. However, the aqueous phase of the butanol extract was purified using a C18 pipette tip and analyzed by mass spectrometry. Results in **Figure 5C** indicate that butanol extraction only isolated a fraction of the fully modified peptide since traces of triple dehydrated products were visibly apparent in the mass spectrum of the aqueous phase extract. Furthermore applying direct C_18_-ZipTip purification of the crude TEV digest also produced a mixture of the two species (**Figure 5D**). Fully dehydrated preRumA^∗^ is schematically represented in **Figure 5E**.

### Mass-Spectrometric Fragmentation Analysis of preRumA^∗^

After demonstrating that the dehydratase domain of RumM can catalyze dehydration of the peptide, subsequent mass-spectrometric experiments were performed to demonstrate the activity of the cyclase domain. The cyclisation reactions introduce thioether cross bridges between dehydrated Thr/Ser residues and the sulfhydryl groups of specified cysteine residues. Such information cannot be derived from the exact masses alone because the dehydrated linear peptide (with no thioether rings) and the cyclic forms (with thioether rings) have identical masses, with a resultant average molecular mass of 5444.27 Da. Although the possibility to digest with trypsin at position -1 exist, MS^2^ data from trypsin digests are not discussed since we obtained better fragmentation data with the full-length peptide.

**FIGURE 6:**
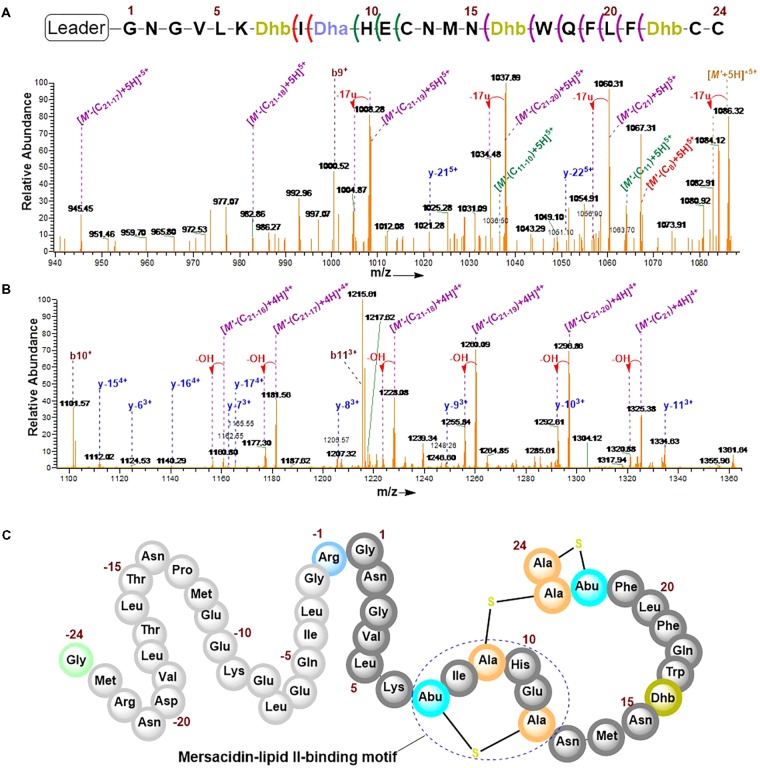
Tandem MS^2^-experiments and assignment of preRumA^∗^ fragment ion peaks. **(A)** and **(B)** nLC-MS^2^ spectra representing ion series with intensive peaks produced as a result of the fragmentation of the C-terminus of preRumA^∗^ core peptide. (*M*’+5H)^5+^ = 1089.72 RumA^∗^. A detailed description of the mass spectra is presented in **Figure 7**. **(C)** Schematic representation of the structure of modified preRumA^∗^; showing the mersacidin-lipid II-binding motif.

**FIGURE 7:**
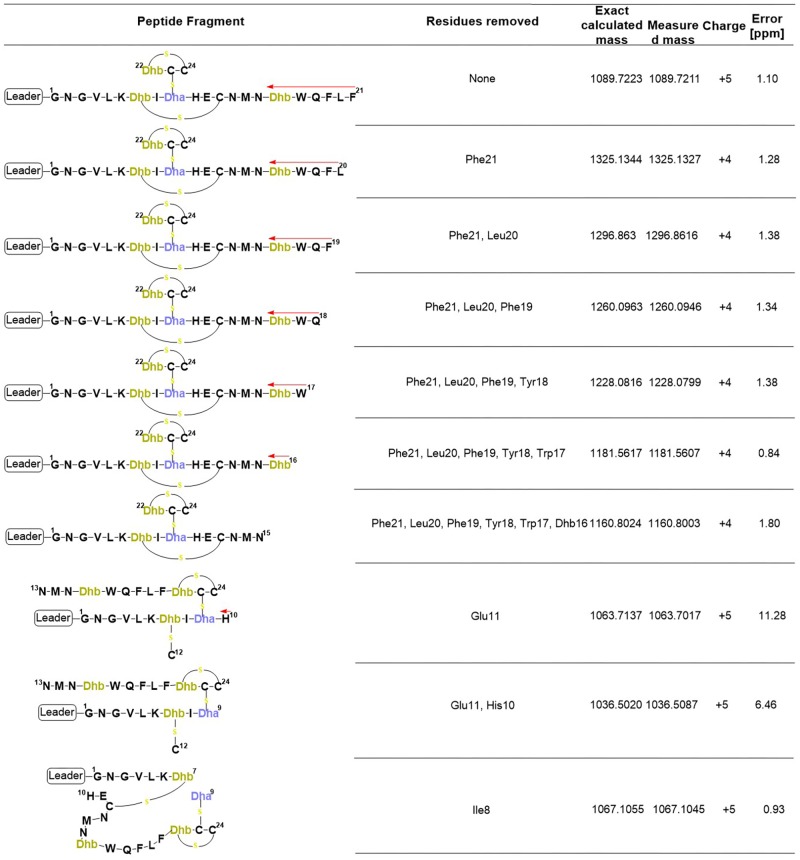
Product ions derived from the fragmentation of the C-terminal core peptide of preRumA^∗^. This figure describes the possible cross-linked structures that gave rise to the product ions identified in the mass spectrum. The charges and measurement precisions in parts per million (ppm) are also indicated.

The combination of collision-induced dissociation (CID), higher-energy collisional dissociation (HCD) and electron transfer dissociation (ETD) at different fragmentation voltages produced different fragmentation pattern (Supplementary Figure S6). The N-terminal leader sequence exhibited b- and y-type ions with higher intensities which nicely fitted to the first 19 residues of the leader peptide (Supplementary Figure S7). Although no intensive b- and y-type ions were visible at the C-terminus, intensive ion series representing fragments corresponding to a successive loss of internal amino acid residues from the C-terminal region were observed (**Figures 6A,B**). These data demonstrate the successive loss of residues 21–16, 11–10, and residue eight of the core peptide (See **Figure 7** and the next paragraph). Note that in assigning the product ion peaks the following notations were considered: *C*_n_ denotes the core peptide residue (*C*) at specific position (*n*); while *C_n_*_-_*_n_*_^′^_ denotes core peptide residues from position *n* to position *n*^′^ following a C- to N-terminal direction. No further intensive fragment ions within the core peptide were identified. This data was consistent with those obtained by Dabard and colleagues, however, fragment ions resulting from the double cleavage at the amino and carboxyl termini of Ile8, His10, and Glu11 altered our understanding of the structural features of fully modified RumA. These ions retained information for all other residues in the peptide sequence even after the residues mentioned were cleaved-off during the fragmentation process. This data demonstrated that bonds other than the peptide bonds were responsible for holding the two adjacent fragments together. This was surprising because data from previous characterization of RumA by Dabard and colleagues did not show this (Dabard et al., 2001). From the data in hand, the structure of modified preRumA^∗^ may be schematically represented as shown in **Figure 6C**.

To further expatiate on the structural description of the various fragments that gave rise to the intensive product ion peaks identified in the mass spectrum, different structures were proposed of which those presented in **Figure 7** fitted precisely to the measured *m*/*z*. Notice that each structure represent an internal double cleavage of the polypeptide backbone from the C-terminus that resulted in product ions associated with the successive loss of amino acids in the direction indicated by the arrow. Any loss of an internal residue should automatically result in b- and/or y-type ion(s) or other associated ion types like a, c, x, and z. Such was not the case observed with the fragmentation of C-terminal preRumA^∗^. Not even the x- and z-type ions which are observed during *de novo* peptide sequencing were identified. Instead, the double cleavage fragments hosted residues present upstream and downstream of the cleavage position. This is only possible if there is a bond linking the C-terminal fragment to the N-terminal segment. The presence of cysteine or Dhb/Dha residue adjacent to these cleavage positions allowed us to allocate thioether cross-linkages. Moreover, **Figure 7** shows details of how the various structures were associated to their respective molecular masses, with very high precisions.

### Iodoacetamide Derivatization and Trypsin Digestion of preRumA^∗^

**FIGURE 8:**
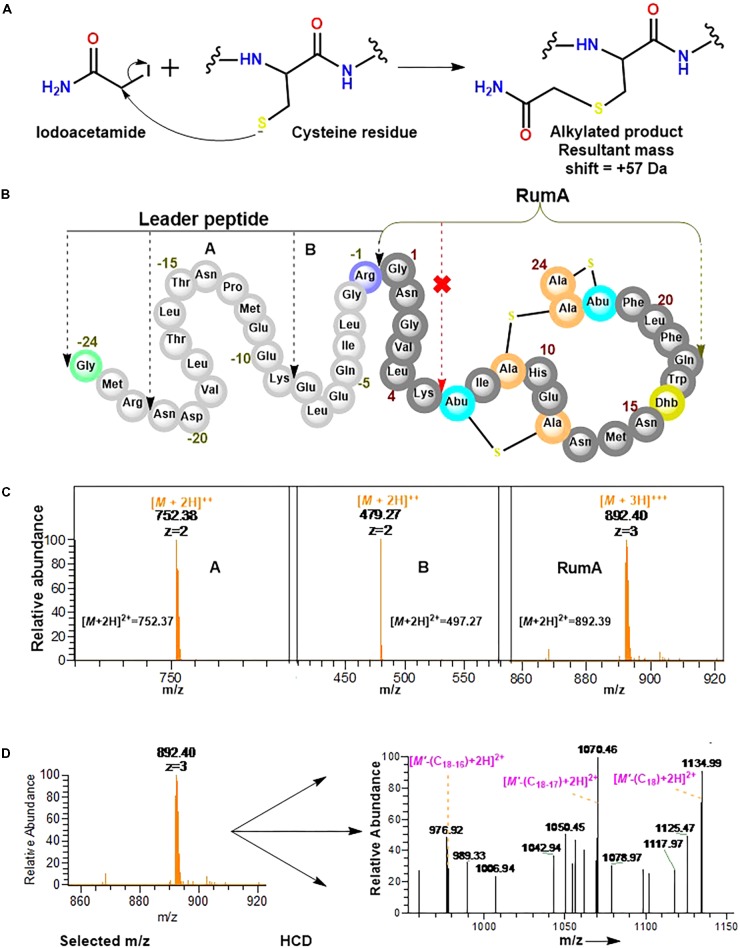
Iodoacetamide derivatization and trypsin cleavage of preRumA^∗^. **(A)** Iodoacetamide reaction with the sulfhydryl group of cysteine side chain. **(B)** Schematic representation of preRumA^∗^ showing five different fragments expected from the tryptic digestion. **(C)** Charged ions peaks observed in the Orbitrap Fusion nLC-MS spectrum of the digested preRumA^∗^ products. The predicted exact masses for each of the fragments are labeled in their respective spectra. **(D)** MS^2^ experiments and assignment of trypsin-activated RumA fragment ion peaks. Major product ions in the spectrum were identified to fit successive loss of Gln18, Trp17 and Dhb16. (*M*’ +3H)^3+^ = 892.40^∗^.

Experiments here were designed to demonstrate that the cysteine residues in the core peptide of preRumA^∗^ were involved in the thioether ring formation and to determine whether or not trypsin can cleave preRumA^∗^ at the carboxyl terminus of Lys6. Our approach here included an iodoacetamide derivatization step followed by an overnight trypsin digestion at 37°C. The iodoacetamide reaction was required to determine if the three cysteine residues present in the core peptide had free sulfhydryl side chains. As described in **Figure 8A**, the thiol group of cysteine undergoes an irreversible reaction with iodoacetamide to produce an alkylated species with a consequent mass shift of +57 Da in the mass spectrum. Interestingly, no charged ionic species was identified with a mass shifts associated to mono-, double-, or triple- carbamidomethylation of the full length nor other fragments labeled in **Figure 8B**.

Assuming that preRumA^∗^ did not contain any Lan/MeLan rings, all three or at least one of the cysteine side chains in the core peptide would have been *S*-carbamidomethylated. This data supplied additional evidence to demonstrate that the sulfhydryl groups of all three cysteine residues in the core peptide were not accessible to the alkylation reaction. Thus all the thiols must have been protected via an alternative reaction. It is therefore reasonable to say that the cysteinyl thiols were involved in the cyclization step of the biosynthesis pathway which rendered them unavailable for any further reaction with iodoacetamide. This was not the case for unmodified His6-preRumA discussed earlier, where all the cysteine residues were alkylated (**Figure 2D**).

Conversely, charged ion species representing specific fragments belonging to the leader sequence as well as full-length core peptide (RumA) were identified (**Figure 8C**). The fact that we were not able to identify fragments pertaining cleavage at Lys6 of the core peptide indicated that this position was not accessible to trypsin. This data supports the findings of Dabard et al. (2001) who showed that PTMs at Thr7 forbid the possibility of trypsin cleavage at the adjacent Lys6 residue. Furthermore, fragmentation of the RumA precursor ion yielded a product ion spectrum with very little information due to low concentration. However, we were able to deduce that some major peaks in the spectrum carried the same information as reported for MS^2^ of the full length species (**Figure 8D**).

### Interactions Between His6-RumM and His6-GFP-TEV-preRumA^∗^

**FIGURE 9:**
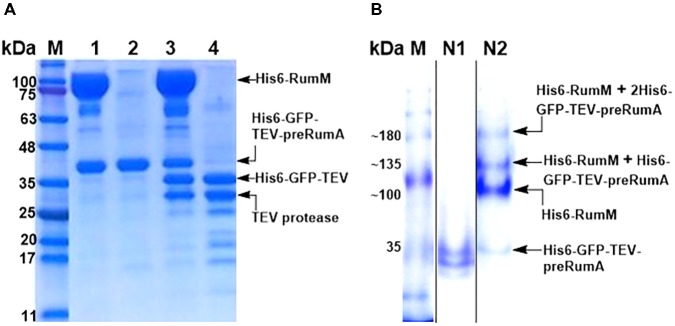
TEV cleavage analyses and Native PAGE. **(A)** SDS-PAGE analysis of gel filtration-purified His6-RumM/His6-GFP-TEV-preRumA^∗^ complex (lane 1), free His6-GFP-TEV-preRumA^∗^ (lane 2), TEV digest of complex (lane 3) and TEV digest of free His6-GFP-TEV-preRumA^∗^ (lane 4). **(B)** Native PAGE analysis of purified His6-GFP-TEV-preRumA from WLEO*grA^∗^* strain (lane N1) and the His6-RumM/His6-GFP-TEV-preRumA complex sample (lane N2). Lane N2 shows multiple bands above the expected molecular weight of His6-RumM corresponding to 1 or 2 molecules of His6-GFP-TEV-preRumA^∗^ complexed to one molecule His6-RumM.

As shown earlier, His6-RumM and His6-GFP-TEV-preRumA^∗^ were coeluted together during size exclusion chromatographic purification despite the huge size difference between the two. We obtained samples from the pooled fractions of representative chromatographic peaks (**Figure 4D**, peaks A^′^ and B^′^) and subjected them to TEV digestion. Results indicated that only about 50% of His6-GFP-TEV-preRumA^∗^ complexed with His6-RumM was digested by TEV whereas the same construct not complexed with His6-RumM was completely digested (**Figure 9A**). This partial susceptibility to TEV suggests possible molecular interactions between His6-RumM and the precursor peptide preRumA^∗^ fused to the C-terminus of GFP, since such interactions may possibly shield the cleavage site that links the two and render it inaccessible to TEV.

Partial resistance to TEV cleavage may be as a result of cooperative/noncooperative interactions between His6-GFP-TEV-preRumA^∗^ and His6-RumM as demonstrated by Khusainov and colleagues for the class I lanthipeptide precursor NisA and its modifying enzyme NisB (Khusainov et al., 2011). If His6-GFP-TEV-preRumA^∗^ and His6-RumM were to interact cooperatively, both the core segment and the leader segment of preRumA^∗^ would distinctively bind to His6-RumM at the same time, and if noncooperative, either one of them would bind. This makes sense because the functions of RumM are similar to that of NisB, with an additional cyclase function which NisB does not perform. This mode of interaction may be used to explain the current observation since noncooperative interaction with the core peptide alone may allow TEV to gain access to the cleavage site as the site is located upstream of the leader peptide. However, noncooperative interaction of RumM with the leader sequence alone, or a cooperative mode which involves both segments may bury the TEV site and render it inaccessible to cleavage. Note that the interactions are not stable and so the sequential bind/release cycles may contribute to slow down the activity of TEV (see last paragraph of the “Discussion” section).

To further verify if there was indeed a complex formation between His6-RumM and His6-GFP-TEV-preRumA^∗^, a sample was obtained from the His6-RumM/His6-GFP-TEV-preRumA coeluted fraction and analyzed with native PAGE, together with purified His6-GFP-TEV-preRumA^∗^ from WLEO*grA^∗^* strain employed as a control. The usual two bands obtained for construct purified from WLEO*grA^∗^* or WLEO*grA* (Supplementary Information section “Purification and TEV Cleavage of preRumA^∗^”) was visibly apparent whereas four distinct bands belonging to His6-GFP-TEV-preRumA^∗^, His6-RumM and complexes with molecular weights above that of RumM were present in sample obtained from His6-RumM/His6-GFP-TEV-preRumA coeluted fraction (**Figure 9B**). It is unlikely that these were just mere aggregation since gel filtration fractions (that were extensively diluted) still contained these complexes (Supplementary Figure S5A). Result here shows that the interactions are reversible since His6-RumM and His6-GFP-TEV-preRumA^∗^ bands were also visible. The outcome further suggests that the complex formation may have occurred between His6-RumM and dehydrated preRumA^∗^ since extracted preRumA^∗^ from the TEV digests all contained fully cyclized product, and Mavaro et al. (2011) already demonstrated that the interaction between modifying enzyme and dehydrated precursor peptide has the highest affinity (Mavaro et al., 2011).

### Bioassay Analysis of Trypsin Activated His6-SUMO-preRumA^∗^

**FIGURE 10:**
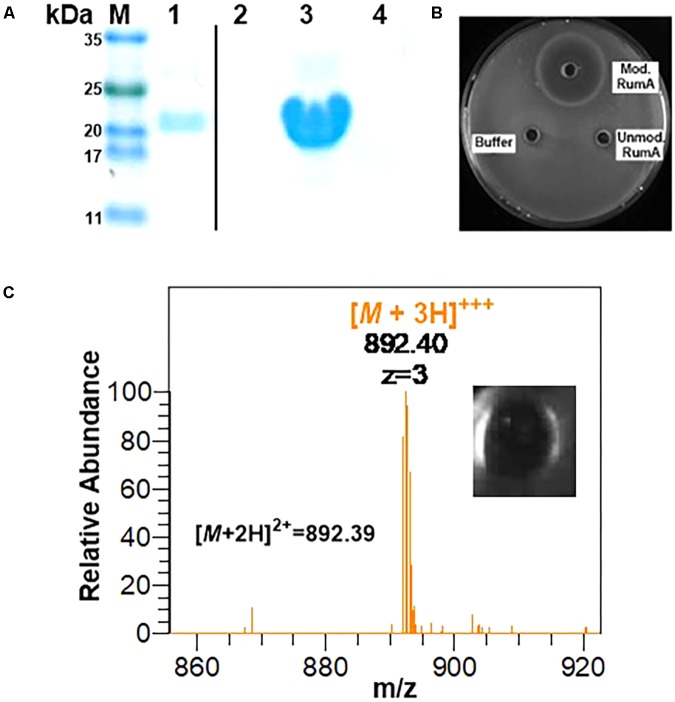
*In vitro* trypsin activation of His6-SUMO-preRumA and bioassay. **(A)** SDS-PAGE analysis of IMAC-purified modified His6-SUMO-preRumA^∗^ (lane 1), trypsin-digested modified His6-SUMO-preRumA^∗^ (lane 2), IMAC-purified non-modified His6-SUMO-preRumA^∗^ (lane 3) and trypsin-digested non-modified His6-SUMO-preRumA^∗^ (lane 4). **(B)** Bioassay analysis of the trypsin digested products of modified and non-modified His6-SUMO-preRumA^∗^ against *B. subtilis ATCC 6633* grown on *a* culture plate. **(C)** Mass spectrum showing prominent ion peak corresponding to active RumA purified from the trypsin digest of cyclized preRumA^∗^ and bioactivity of the resulting product.

Using GFP as a fusion partner may also compromise the overall yield of the final product because GFP itself is about five times larger than the precursor peptide preRumA. To mitigate this effect, we tested another fusion partners like the SUMO. Two plasmids were designed to fuse preRumA^∗^ to SUMO (Supplementary Figure S1). Purified His6-SUMO-preRumA^∗^ from the two strains WLEOsrA^∗^ (expressing His6-SUMO-preRumA^∗^) and WLEOsrA^∗^M (expressing His6-SUMO-preRumA^∗^ and His6-RumM) were digested with trypsin (**Figure 10A**). His6-SUMO-preRumA^∗^ digest from the latter strain was expected to yield putative mature ruminococcin-A. The digested products were pipetted into wells created in an agar plate spread with *B. subtilis ATCC 6633* and subsequently incubated at 30°C overnight. A distinct zone of inhibition was visible for the modified His6-SUMO-preRumA^∗^ construct and none for the unmodified construct as well as trypsin digestion buffer that was used as a control (**Figure 10B**) suggesting that only the modified product has growth inhibitory biological activity. Additionally, the tryptic digest obtained from the *in vitro* activation of preRumA^∗^ (derived from WLEO*grA^∗^M1*) was also pipetted onto an agar plate spread with *B. subtilis ATCC 6633*. A distinct zone of inhibition was observed (**Figure 10C**), suggesting that the isolated peptide also possessed growth inhibitory biological activity against *B. subtilis ATCC 6633.*

Although we did not characterize products obtained from the SUMO-fused constructs, being able to observe growth inhibitory activity in extracts isolated from a system expressing both preRumA and RumM and no activity in those isolated from the system expressing preRumA alone, allowed us to conclude that the SUMO fusion construct is also functional.

## Discussion

In the present study, we have shown that fusing the structural gene for the precursor peptide preRumA to the gene encoding the fast folding GFP and co-expressing the chimeric construct simultaneously with the ruminococcin-A lanthionine synthetase RumM resulted in the biosynthesis and modification of the peptide *in vivo*. These results demonstrate that in a heterologous host like *E. coli*, a larger attachment to the N-terminus of leader peptide does not obstruct *in vivo* processivity of RumM in catalyzing formation of PTMs in the core peptide of preRumA. To the best of our knowledge, this is the first illustration which demonstrate that the precursor peptide can still be correctly modified with such a large fusion partner attached to the leader sequence. This therefore provides an alternative approach to derive some insights into mechanistic events underlying the generation of MeLan/Lan rings as well as dehydroamino acid in lanthipeptides. Thus the system developed herein has potentials to be utilized in a subsequent study as a tool to gain more insights into the catalytic mechanisms of LanM enzymes.

We were able to obtain approximately 6 mg of modified preRumA^∗^ per litee of *E. coli* culture (**Table 2**). Considering the size ratio of leader-to-core segment (∼1:1.02), this amount may be factored down to approximately 1–2 mg of pure and active ruminococcin-A per liter of culture if the leader peptide is removed. The nature of *R. gnavus* E1 poses several production challenges with respect to growth and optimization. Dabard et al. (2001) only succeeded in achieving a yield of 0.665 μg of RumA per liter of *R. gnavus* E1 culture (Dabard et al., 2001). The specific activity of RumA would have been a more appropriate parameter to describe the quality of the expression and purification procedures. However, the main focus of this study was first to show that it is possible to produce this peptide in *E. coli*, characterize its structure, show that it is active and then set the platform for strain optimization. Additionally, TEV cleavage released the fully modified precursor but not the active product. The product only becomes active once the leader peptide is removed. Our strategy to remove the leader peptide involved the use of trypsin. There are three active trypsin cleavage sites in preRumA^∗^ and therefore, a trypsin cleavage reaction will result in a mixture of four different fragments from which to isolate the active RumA. Further investigations would include introducing a unique cleavage site (e.g., Factor Xa) between the leader and the core segments to facilitate purification of the active product. We made unsuccessful attempts to introduce a TEV cleavage site at this position, probably because the cleavage sequence is too long and so did not allow efficient modification of preRumA by RumM. Nevertheless, activity against *B. subtilis ATCC 6633* was observed in crude tryptic digests of preRumA^∗^ and His6-SUMO-preRumA^∗^, which were enough to show that the peptide is active.

It is also unlikely that regulating cultivation parameters and engineering cultivation media would generate a meaningful improvement with regards to product titres. Even if that would be the case, the cost of production would be exceedingly high with respect to the additional costs incurred by supplying trypsin as a supplement to the cultivation medium. We have succeeded to demonstrate that it is possible to produce RumA in *E. coli* with higher yields, presenting an efficient strategy to consistently develop a biotechnological process for the production of the AMP. In fact, the numbers reported herein overwhelmingly exceed the amount of RumA purified from the native host in the order of 10^4^, but comparatively lower with respect to other reported cases (Shi et al., 2011, 2012; Tang and van der Donk, 2012; Kuthning et al., 2015). However, this can be improved by performing further optimization including strain engineering or employing the use of smaller fusion partners like SUMO or thioredoxin. Nevertheless, for the purpose of studying peptide engineering and LanM catalysis, the current system represents a perfect tool. Additionally, other technologies which aim at expanding and/or increasing antimicrobial activity of the compound, like the use of amber stop codon suppression to incorporate non-canonical amino acids into the peptide (Steiner et al., 2008; Chatterjee et al., 2012; Oldach et al., 2012) may be easier to apply with the present system.

With respect to structural characterization of modified preRumA^∗^, data presented herein are consistent with those obtained by Dabard and coworkers, with the exception of one additional methyllanthionine ring between Thr7 and Cys12 which was never reported. In fact, Dabard et al. (2001) concluded in their study that the MeLan ring formation between these two residues was not possible. Nevertheless, they arrived at this conclusion using information derived from Edman degradation assay. However, the Edman sequencing method is not very accurate in studying the structure of lanthipeptides because of limitations associated with the fact that any modification at the N-terminus of the peptide sequence will block the experiment. Additionally, Edman degradation is blocked by Lan/MeLan rings as well as Dhb/Dha residues, and no sequence information can be obtained thereafter (Lohans and Vederas, 2014). Based on our mass spectrometry studies and data obtained from the iodoacetamide derivatization reaction, we may conclude with certainty that the structure described in **Figure 6C** represents the actual structure of the modified precursor peptide. Additionally, the third ring identified herein also constitutes the core active feature referred to as the mersacidin-lipid II-binding motif which is common in all class II lanthipeptides (Knerr and van der Donk, 2012).

This implies that in *E. coli*, the dedicated ruminococcin-A lanthionine synthetase RumM is capable of catalytically installing three thioether cross-linkages and one additional α,β-unsaturated amino acid Into the core structure of preRumA. The dehydratase domain of RumM catalyzes the dehydration of Thr7, Thr16, and Thr22 to three Dhb, and Ser9 to Dha. Subsequently, the cyclase domain engages in a Michael-type addition-cyclisation reactions involving Dhb7 and Dhb22, and activated thiol groups of Cys12 and Cys24 to produce two MeLan rings, while the Dha9 and Cys23 produce a Lan ring.

**FIGURE 11:**
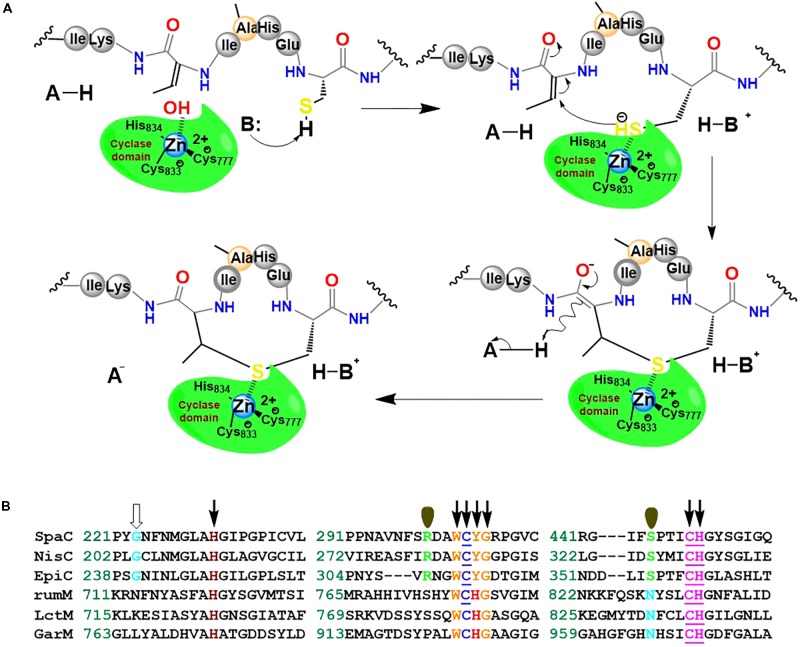
Proposed mechanism of Michael-type addition reaction catalyzed by RumM. **(A)** The C-terminus of the bifunctional enzyme possesses the conserved residues that have been shown to coordinate Zn^2+^ ion in the active site of some lanthionine cyclase. The scheme illustrates the formation of the first N-terminal lanthionine ring of RumA. **(B)** Highly conserved domains of selected lanthionine cyclases. The underlined residues are responsible for coordinating the central Zn^2+^ ion as shown in the crystal structure of NisC described earlier (Li et al., 2006; Li and van der Donk, 2007). The residues indicated by arrows are essential for an active modification of subtilin, whereas those indicated by single orbitals appear to have no effect on SpaC catalytic activity (Helfrich et al., 2007). The conserved Gly residues denoted by an open arrow was shown to be highly implicated in the maturation of epidermin (Agustin et al., 1992).

We hypothesize that RumM-catalyzed Michael-type addition cyclisation reactions may follow the scheme proposed in **Figure 11A**. The scheme is similar to the mechanism proposed elsewhere for nisin cyclase (Li et al., 2006). Although low sequence identity exist across the family members of lanthionine-generating enzymes, the C-terminus of RumM possesses conserved active site residues identical to those of nisin cyclase (**Figure 11B**), indicating possible structure-function similarities, and hence we postulate that their mode of action may be closely similar. This type of structure-function similarity has been described in a variety of the lanthionine-generating enzymes (Knerr and van der Donk, 2012; Repka et al., 2017). The scheme proposes that dedicated active site residues of the cyclase domain of RumM may coordinate a hydrated Zn^2+^ cofactor whose water molecule could be displaced by a cysteinyl thiol (e.g., Cys12 of preRumA) which is targeted for conjugation with a Dhb/Dha in the core peptide (e.g., Dhb7 of preRumA). By so doing, the thiol group by itself may become activated, creating a nucleophilic active center. The displacement of the water molecule or the activation of the cysteinyl thiol may be facilitated by His778 which probably play the role of an acid/base catalyst like the case of Tyr304 in SpaC (Helfrich et al., 2007). The electrophilic carbon atom of the Dhb7 may now launch an attack on the activated Cys12, forming an enolate intermediate which may undergo subsequent protonation to generate the thioether cross bridge (**Figure 11A**). Mutagenesis studies in conjunction with mass spectrometry and/or bioassay analyses may be employed to characterize the C-terminal domain of RumM, or perhaps the crystal structure of the full-length enzyme may be more resourceful.

In this study, we initially considered using two plasmids to separately control the biosynthesis of the precursor peptide preRumA and its modifying enzyme RumM. We considered on the one hand that it was necessary to place *rumM* under the control of a weaker promoter since its product is larger (∼107 kDa) and thus may require more resources for its biosynthesis and a slower process from transcription to translation for the protein to be properly folded and soluble. On the other hand, strong overexpression of the precursor peptide may have little physiological effect on the host since it is relatively small (5.34 kDa). As such, this strategy may help to conceal the effects of cellular proteases on the small peptide thereby increasing cellular availability of the unmodified substrate and the chances for RumM to catalyze PTMs in preRumA. It is challenging to say exactly why this strategy did not generate the expected modifications in preRumA when His6-preRumA was coexpressed with His6-RumM, although Basi-Chipalu and coworkers employed a similar approach to modify pseudomycoicidin in *E. coli* (Basi-Chipalu et al., 2015). We may anticipate that the enhanced solubility (and no modification) of preRumA in the strain WLEO*rA/M* would be facilitated by a simple binding interactions between His6-preRumA and His6-RumM since interactions between non-modified peptide precursor and its modifying enzyme have been reported (Lubelski et al., 2009; Khusainov et al., 2011; Mavaro et al., 2011; Repka et al., 2017).

Furthermore, a mixture of modified and non-modified preRumA were identified in extract obtained from strain WLEO*grA/M* (Supplementary Figures S4C,D) and the presence of non-modified preRumA was not apparent in extract from strain WLEO*grA* (expressing His6-GFP-TEV-preRumA alone), but rather truncated products (Supplementary Figures S4A,B). These outcomes enabled us to reason that RumM may have fostered some form of molecular interactions that in turn conferred a stabilizing role on the precursor peptide since it is believed that the enzyme forms a complex with the precursor peptide (Lubelski et al., 2008, 2009). Data also exist, suggesting a constant interaction with both the non-modified and the modified substrate (Yang and van der Donk, 2015). These results further supported our hypothesis that biophysical interactions between His6-preRumA and His6-RumM may have facilitated the solubility of the former without necessarily introducing the desired modifications in the peptide. Nevertheless, mixtures of partially dehydrated products are possible since investigations revealed that the coupling of cysteinyl thiol to Dha/Dhb can prevent further modification of serine and/or threonine residues (Kuipers et al., 2008; Lubelski et al., 2009). We cannot at this time report whether the intermediate products observed contained at least one of the rings or not. Nevertheless, our results show that low levels of RumM may have been responsible for the inefficiency of strain WLEO*grA/M*.

The amount of enzyme produced by the weakly expressing vector may not be optimal for complete modification of the precursor peptide to be achieved, which may explain why unmodified preRumA was identified in extract from the WLEO*grA/M* strain. This appeared to be the case as this drawback was mitigated by reengineering the host vector to favor adequate production of RumM. It is important to note that applying the single-plasmid bicistronic expression vector system did not improve the quality of His6-GFP-TEV-preRumA when RumM was expressed under the control of the *CU* promoter and RBS from the lactose operon (data not shown). However, by replacing the RBS from the lactose operon with that of gene 10 of bacteriophage T7, RumM expression was enhanced and the degradation problem was solved. In fact, the strain WLEO*grA^∗^M1* produced only minute traces of threefold dehydrated peptide and no unmodified peptide compared to strain WLEO*grA/M* (Supplementary Figure S8). Therefore, it is important to ensure adequate expression of the modifying enzyme when trying to develop a combinatorial system for the production of lanthipeptides in *E. coli.*

The interactions observed between His6-GFP-TEV-preRumA^∗^ and His6-RumM may be further investigated to supply more insights into the nature of complexing. Crystallization experiments may be worthwhile to characterize these complexes, which may reveal some structural features in LanM enzymes yet to be known. Our data suggests that dehydrated preRumA^∗^ may be predominant in the His6-GFP-TEV-preRumA^∗^**/**His6-RumM complex since it has been reported that the dehydrated precursor peptide binds the modifying enzyme with highest affinity compared to the active and the unmodified forms (Mavaro et al., 2011). Supposing that the interactions between the precursor peptide and the modifying enzyme does not form a stable complex, and the fact that a LanM enzyme was recently shown to catalyze reversible opening of the thioether rings (Yang and van der Donk, 2015), two key points may be highlighted in the present case. (1) RumM may slowly catalyze ring opening of the fully cyclized preRumA^∗^ thereby supplying the dehydrated precursor to the reaction milieu. (2) Since the formation of thioether ring is an exergonic process that favors closed-ring conformations in the peptide over the opened-ring forms (Krenske et al., 2011), the dehydrated precursor may immediately form a complex with RumM to facilitate thioether ring formation again. Whether or not the reaction medium can permit this is left for further investigation. Nevertheless, the fact that preRumA^∗^ extracted from the TEV digests all contained thioether rings indicated that majority of the cleavage product was the fully cyclized peptide—the active peptide essentially shows negligible binding affinities to the enzyme as demonstrated by Mavaro et al. (2011).

## Conclusion

In this study, we succeeded in producing fully modified ruminoccin-A in *E. coli*. The modified precursor preRumA^∗^ (containing a Gly-1/Arg mutation) was activated *in vitro* by cleaving off the leader peptide using trypsin. *In vivo* bioactivity against *B. subtilis ATCC 6633* was achieved using crude tryptic digests of preRumA^∗^. Structural and molecular characterization supplied further insights into the nature of modifications in RumA and the mode of catalysis involving LanM enzymes. We show that mature RumA contains three lanthionine cross bridges instead of two as previously reported. We achieved approximately 6 mg of modified preRumA per liter of *E. coli* culture, which corresponds 1–2 mg of final active product, a yield improvement of about 10^4^ compared to yields obtained from the native producer (Dabard et al., 2001). The system developed in this work offers an irrefutable advantage over the native producer in that the essential genes can be optimally controlled to allow improved production. In a subsequent investigation, the systems developed herein may be applied as a useful tool for mutagenesis investigations and scale-up studies to achieve economic feasibility since complicated and expensive experimental set-ups would not be necessary.

## Author Contributions

EO and PN conceived the study. EO and RG designed and analyzed the experiments. EO conducted the experiments. MF analyzed and validated bioassay. EO and LA did the mass-spectrometric analyses and data evaluation. JR analyzed and evaluated MS data from His6-preRumA. All authors wrote the manuscript.

## Conflict of Interest Statement

The authors declare that the research was conducted in the absence of any commercial or financial relationships that could be construed as a potential conflict of interest.

## Footnotes

1 http://spectrumviewer.org/
