# Supplementary material for: Heterologous Biosynthesis, Modifications and Structural Characterization of Ruminococcin-A, a Lanthipeptide From the Gut Bacterium Ruminococcus gnavus E1, in Escherichia coli
Source: Front Microbiol. 2018 Jul 26;9:1688. doi: 10.3389/fmicb.2018.01688 (PMC6071512; doi:10.3389/fmicb.2018.01688)
Supplement: Supplementary file 1 [file Data_Sheet_1.PDF]

Supplementary Material

Heterologous biosynthesis, modifications and structural characterization of ruminococcin-A, a lanthipeptide from a gut bacterium, in *Escherichia coli*

Elvis L. Ongey*, Robert Giessmann Michel Fons, Juri Rappsilber, Lorenz Adrian and Peter Neubauer

*** Correspondence:** Corresponding Author: elvis.ongey@gmail.com

***Correspondence address**

[1]Chair of Bioprocess Engineering

Institute of Biotechnology

Technische Universität Berlin

Ackerstraße 76, ACK24

D-13355 Berlin, Germany

Tel.: +493031472269

Fax: +493031427577.

1. Construction of Expression Vectors for *E. coli* Production

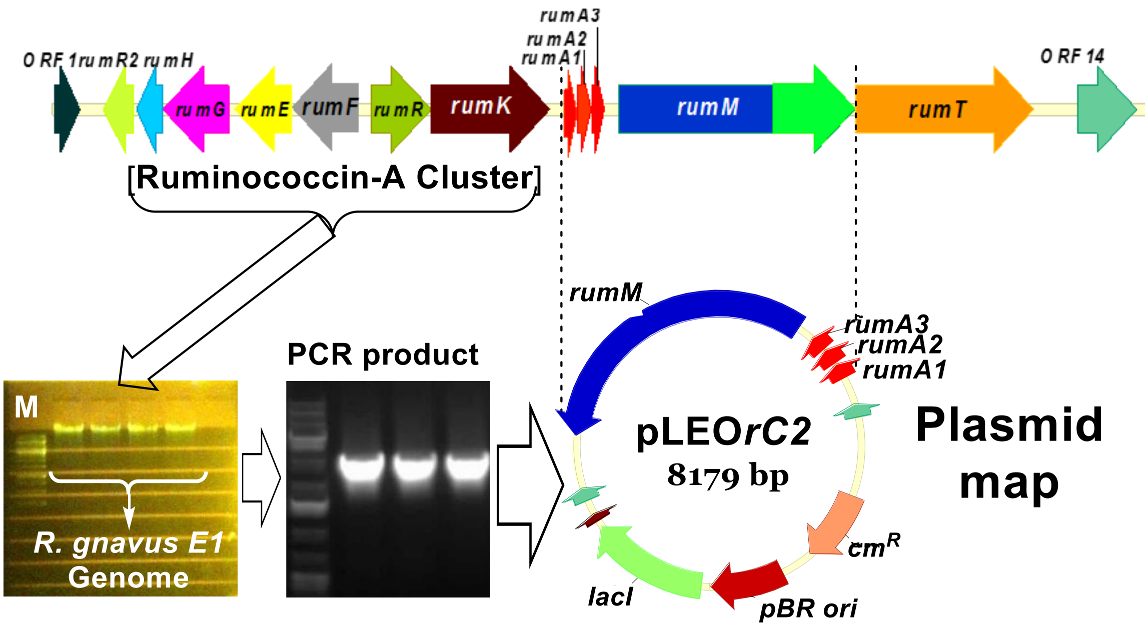

Supplementary Figure 1 | Procedures used for isolating and cloning cluster fragment containing the *rumA* genes and *rumM*. Purified *Ruminococcus gnavus E1* genome was used as template for PCR.

Supplementary Table 1 | Oligonucleotides used in this work

	Name_ orientation		Oligonucleotide sequence	
1	*rumClus_f*	5´-	ATGAGAAATGATGTATTAACATTAACAAAC…………………………………....	3´
2	*rumClus_r*	5´-	TTACACAGTTTCAAGCATTAAAACAGAG……………………………........	3´
3	*rumA_f*	5´-	AACAGCTAGCATGAGAAATGATGTATTAAC………………………………	3´
4	*rumA_r*	5´-	TGAGTCTGCAGTTAGCAGCATGTAAACAG……………………………….....	3´
5	*rumM_f2*	5´-	TCGA GCTAGC ATGCATAAGAAGTTTTGTGG…………………………………	3´
6	*rumM_f3*	5´-	TCGAGGATCCATGCATAAGAAGTTTTGTGG…………………………………….	3´
7	*rumM_r*	5´-	AAGTCTGCAGTTACACAGTTTCAAGCATTA…………………………………….	3´
8	*gfp_f*	5´-	TAACAGCTAGCATGAGTAAAGGAGAAGAAC	3´
9	*gfp‡_r*	5´-	GTTAATACATCATTTCTCATGCCCTGAAAATACAGGTTTTCTTTGTATAGTTCATC CATG	3´
10	*rumA‡_f*	5´-	GAA AAC CTG TAT TTT CAG GGC ATGAGAAATGATGTATTAAC……………....	3´
11	*pTrypsin_f*	5´-	GATCTTGGGTagaGGTAATGGTG……………………………………………….	3´

12	*pTrypsin_r*	5´-	TGCTCCAGTTCTTTTTCTTC…………………………………………………………	3´
13	*prumM_f*	5´-	AACA CTGCAG AACGTTACTGGTTTCACATTC……………………………………	3´
14	*prumM_r*	5´-	AGT AAGCTT TTACACAgTTTCAAgCATTAAAAC……………………………...........	3´
15	*SDT7_f*	5´-	TTGTACTAGTTCACTGCCCGCTTTCCAG…………………………………………..	3´
16	*SDT7_r*	5´-	GAGTCATATGTATATCTCCTTCTCTAGAAATTCGTAATC………………………..	3´

Forward primer, *f*; reverse primer, *r*; splicing primers ‡

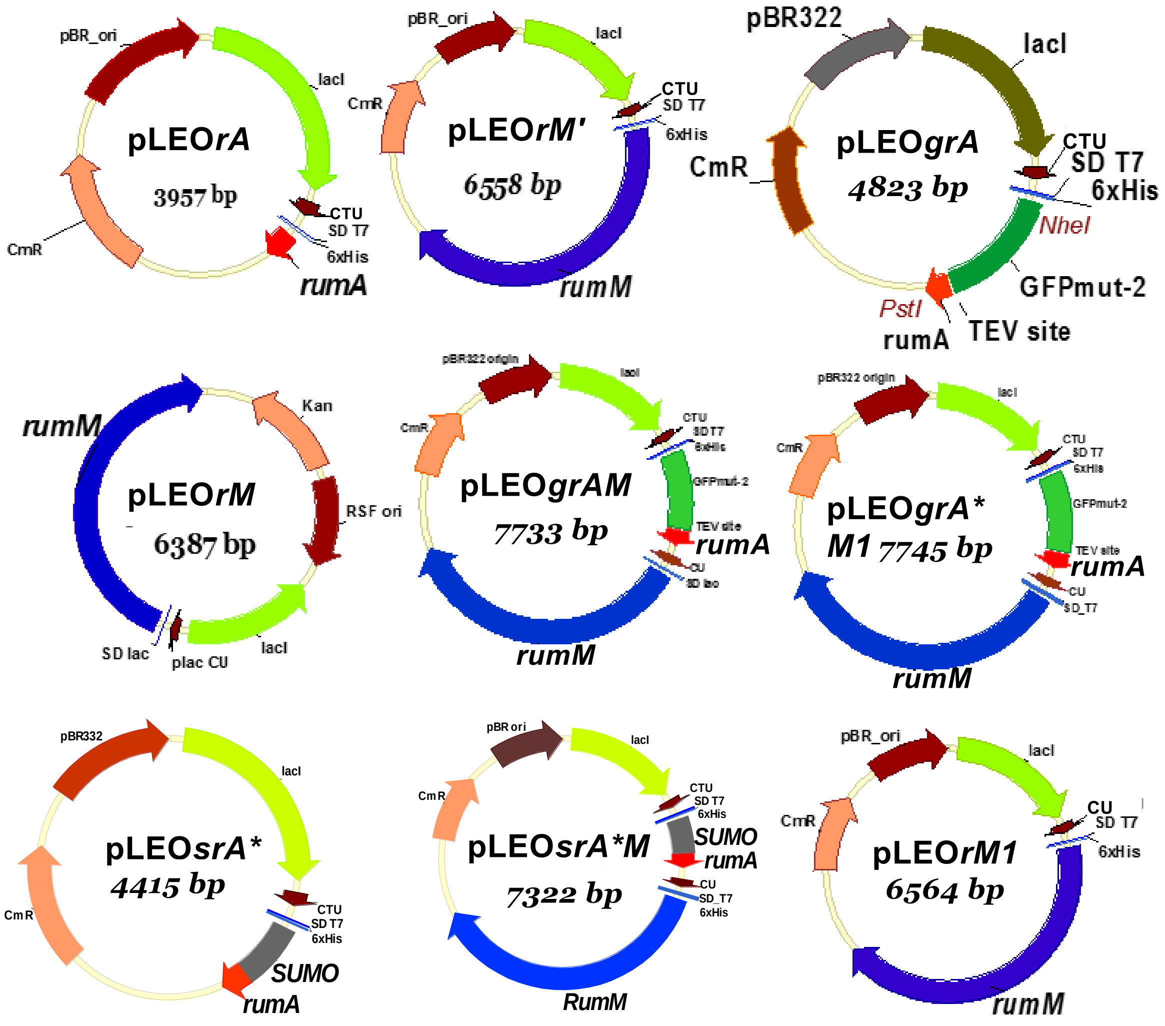

Supplementary Figure 2│Expression vectors description. Maps of expression plasmids indicating all necessary features

2. Fusion of preRumA to GFP, expression and LC-ESI-MS Analyses

Expression of the resultant strains WLEO*grA* (expressing His6-GFP-TEV-preRumA alone) and WLEO*grA/M* (expressing both His6-GFP-TEV-preRumA and His6-RumM on separate plasmids) were monitored online in a 96-well flat-bottom plate via GFP fluorescence signal intensities. The cultures were induced with 100 µM IPTG and time-course fluorescence signal intensities were measured. In all cases, the RFU of the control strain (WLEO*rM'*; expressing His6-RumM only) remained constant throughout the entire cultivation period meanwhile a dramatic drop in the fluorescence signals of WLEO*grA/M* strain was observed (Supplementary Figure 3A). This was also reflected in the amount of purified product obtained from this strain. A possible reason for this may the metabolic burden imposed by the presence of two distinct plasmids. Purified His6-GFP-TEV-preRumA construct from WLEO*grA* and WLEO*grA/M* strains indicated dissimilar migration properties on SDS-PAGE (Supplementary Figure 3B), clearly indicating that His6-RumM has an effect on the His6-GFP-TEV-preRumA construct. All four bands indicated in Supplementary Figure 3B were excised and identified via MS to have completely different primary sequence structures. We were unable to identify full length His6-GFP-TEV-preRumA in both bands 1, 2 and 4. Band 3 appeared to be more interesting and so we decided to investigate further.

IMAC-purified His6-GFP-TEV-preRumA from both systems were dialyzed and cleaved with TEV protease. The digested products together with the non-cleaved samples were analyzed on SDS-PAGE. Interestingly, the expected cleaved product (preRumA) band was not observed in sample purified from strain expressing His6-GFP-TEV-preRumA alone (WLEO*grA*), as opposed to that purified from WLEO*grA/M* which showed a clear band around the expected molecular weight of preRumA (Supplementary Figure 3C). The disparity in their mobility on the SDS gel and the absence of preRumA band in the TEV-digested sample purified from WLEO*grA* supplied the first evidence of the activity of His6-RumM on the GFP fusion construct.

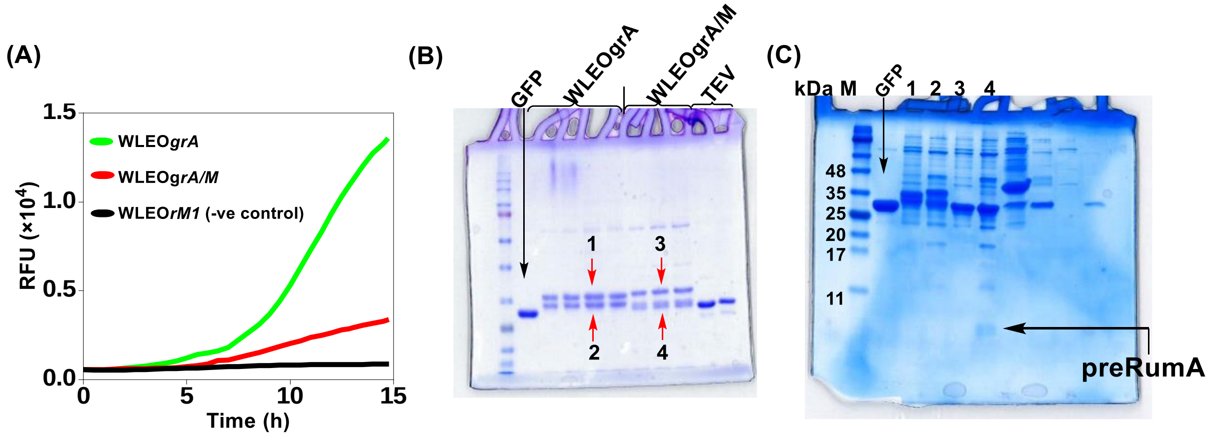

Supplementary Figure 3│Expression of His6-GFP-TEV-preRumA*. **(A)** Time-course GFP fluorescence signals of WLEO*grA*, WLEO*grA/M* and WLEO*rM'* strains cultivated under the same growth conditions. **(B)** SDS-PAGE analyses of purified His6-GFP-TEV-preRumA* from WLEO*grA* and WLEO*grA*/M. **(C)** SDS-PAGE analyses of IMAC-purified GFP, His6-GFP-TEV-preRumA from WLEO*grA* (lane 1), His6-GFP-TEV-preRumA from WLEO*grA/M* (lane 2), TEV-digested His6-GFP-TEV-preRumA from WLEO*grA* (lane 3) & TEV-digested His6-GFP-TEV-preRumA from WLEO*grA/M* (lane 4).

The TEV-digested samples were extracted with 1-butanol and the extracts were dried and re-dissolved in acetonitrile (ACN)/H_2O/formic acid (50:50:0.2%). LC-ESI-MS analysis of extract from the WLEO*grA* system yielded no charged ion peaks corresponding to non-modified preRumA precursor peptide, which further corroborated results obtained from SDS-PAGE (Supplementary Figure 3C). Major peaks resolved in the reversed-phase chromatogram corresponded to truncated preRumA fragments (Supplementary Figure 4A and Supplementary Figure 4B), indicating that the peptide may have undergone host protease degradation.

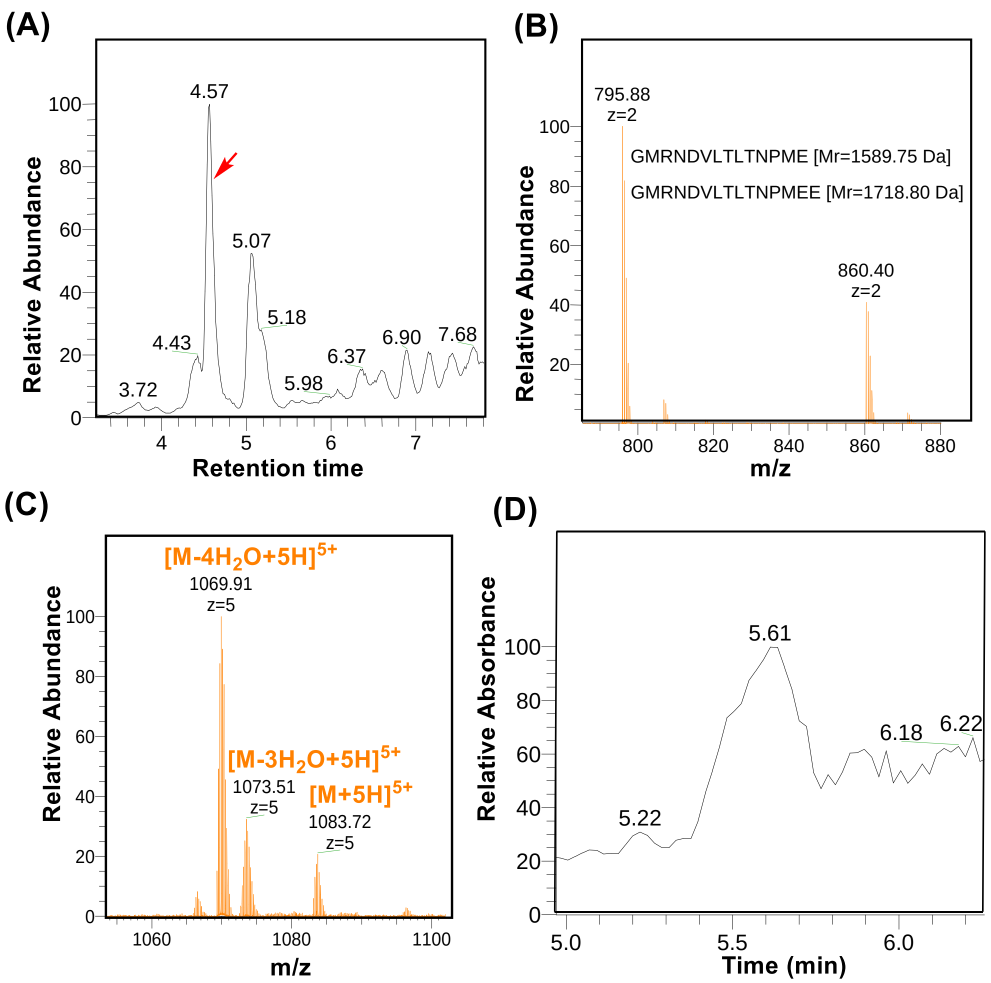

Supplementary Figure 4 | 1-Butanol extraction and LC-ESI-MS. **(A)** Reversed-phase chromatogram obtained from an HPLC run for preRumA extract from WLEO*grA*. The peak indicated by the red arrow corresponds to the mass spectrum in **(B)**. The sequences labelled in **(B)** are degradation or truncated products of preRumA. **(C)** ESI mass spectrum showing mixture of fourfold dehydrated (5344.55 Da), threefold dehydrated (5362.55 Da) and non-modified preRumA (5413.60 Da). The measured exact masses are consistent with the calculated exact masses of 5341.54, 5362.50 and 5413.55 respectively, with a mass error of ± 0.05 Da. **(D)** Reverse-phase chromatogram showing the retention time at which preRumA was eluted.

Interestingly, analysis of extract obtained from WLEO*grA/M* yielded a mixture of 4-fold dehydrated, triple-dehydrated and non-modified preRumA. The latter precedes fully dehydrated preRumA (retention time around 5.51-5.8 min), appearing at a retention time around 5.3-5.5 min

(Supplementary Figures 4C and 4D). The calculated exact molecular mass of non-modified preRumA precursor is 5413.54 Da and the measured value was 5413.60 Da, representing a mass error of + 0.06 Da. Accordingly, 5344.55 Da was measured for the fully modified preRumA, which is also consistent with the expected exact mass of 5341.54 Da, representing a mass error of ± 0.01 Da. These results supply enough evidence to demonstrate that at least dehydration of preRumA is achievable in *E. coli*.

3. Purification and TEV cleavage of preRumA*

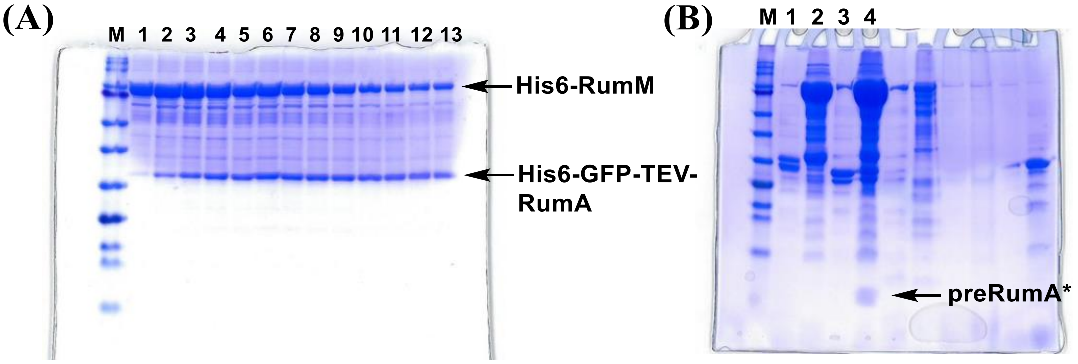

Supplementary Figure 5 | Purification and TEV cleavage. **(A)** SDS-PAGE analysis of individual fractions that constitute the chromatographic peak A' observed during the purification of extracts from strain WLEO*grA*M1*, indicating that both His6-RumM and His6-GFP-TEV-preRumA* were coeluted. Lane 1 shows pure His6-RumM, but from lane 2 a mixture of both His6-RumM and His6-GFP-TEV-preRumA* was apparent. Notice that the amount of His6-GFP-TEV-preRumA* stays fairly constant from lane 4 to around lane 9 while the amount of His6-RumM decreases within this range. Also notice that one of the two bands reported for His6-GFP-TEV-preRumA in the previous section (the lower band) has disappeared **(B)** SDS-PAGE analysis of purified His6-GFP-TEV-preRumA* from strain WLEO*grA** (lane 1) and strain WLEO*grA*M1* (lane 2); as well as TEV digested product of purified His6-GFP-TEV-preRumA* from strain WLEO*grA** (lane 3) and purified His6-GFP-TEV-preRumA* from strain WLEO*grA*M1* (lane 4).

4. The Accuracy of m/z Ratios Measured from Charged Ions

Supplementary Table 2 | Predicted and empirical molecular masses measured in the LC-ESI-MS and Orbitrap Fusion nLC-ESI-MS & MS^2 spectra of *in vivo E. coli* synthesized preRumA.

Wild-type preRumA

Molecular mass[c]	Formula	Exact mass calculated	Mass found	Error [ppm]	Charge
M	C232H369N63O74S6	1083.7143	1083.7201	5.35	5
M-3H2O	C232H363N63O71S6	1073.5085	1073.512	3.26	5
M-4H2O	C232H361N63O70S6	1069.9064	1069.9128	5.98	5
**preRumA***					
M	C236H378N66O74S6	1104.1208	-	-	-
M-3H2O	C236H372N66O71S6	1093.3244	1093.3249	0.45	5
M-4H2O	C236H370N66O70S6	1089.7223	1089.7211	1.10	5
**preRumA* [b-ions]**					
$b3^+$	C13H25N6O3S	345.178	345.1775	1.44	1
$b4^+$	C17H30N8O5S	459.2131	459.2125	1.30	1
$b5^+$	C21H35N9O8S1	574.2401	574.2392	1.56	1
$b6^+$	C26H44N10O9S1	673.3085	673.3075	1.48	1
$b7^+$	C32H55N11O10S2	786.3925	786.3916	1.14	1
$b8^+$	C36H62N12O12S1	887.4402	887.4412	1.12	1
$b9^+$	C42H73N13O13S1	1000.5243	1000.5228	1.49	1
$b10^+$	C46H80N14O15S1	1101.5717	1101.5712	0.45	1
$b11^+$	C50H86N16O17S1	1215.6147	1215.6128	1.56	1
$b12^{2+}$	C55H93N17O18S1	656.8374	656.8366	1.21	2
**preRumA* [y-ions]**					
$y\text{-}23^{3+}$	C234H367N65O69S6	1796.5264	1796.5266	0.11	3
$y°\text{-}22^{3+}$	C229H357N64O67S5	1747.8464	1747.8414	2.86	3
$y°\text{-}21^{3+}$	C223H343N59O67S5	1695.4767	1695.4703	3.77	3
$y\text{-}20^{3+}$	C219H340N58O65S5	1662.7982	1662.7937	2.70	3
$y°\text{-}19^{3+}$	C215H333N57O61S5	1619.12	1619.1201	0.06	3
$y°\text{-}18^{3+}$	C210H324N56O60S5	1586.0968	1586.0925	2.71	3
$y\text{-}17^{3+}$	C204H315N55O60S5	1553.7383	1553.7401	1.15	3
$y\text{-}16^{3+}$	C200H308N54O58S5	1520.0557	1520.0538	1.24	3
$y\text{-}15^{3+}$	C194H297N53O57S5	1482.361	1482.3607	0.20	3
$y\text{-}14^{3+}$	C190H290N52O55S5	1448.6784	1448.6804	1.38	3
$y\text{-}13^{3+}$	C186H284N50O53S5	1410.664	1410.6622	1.27	3

y-12^{3+}	C181H277N49O52S5	1378.313	1378.3112	1.30	3
y-11^{5+}	C176H268N48O51S4	1334.6329	1334.6313	1.19	3
y-10^{5+}	C171H261N47O48S4	1292.6181	1292.6145	2.78	3
y-9^{5+}	C166H254N46O45S4	1248.2709	1248.2687	1.76	3
y-8^{5+}	C160H242N44O44S4	1205.5726	1205.5714	0.99	3
y-7^{5+}	C155H235N43O41S4	1162.5584	1162.557	1.20	3
y-6^{5+}	C149H224N42O40S4	1124.5298	1124.5311	1.15	3

7. Orbitrap Fusion Tandem MS^2 Analyses of PreRumA*

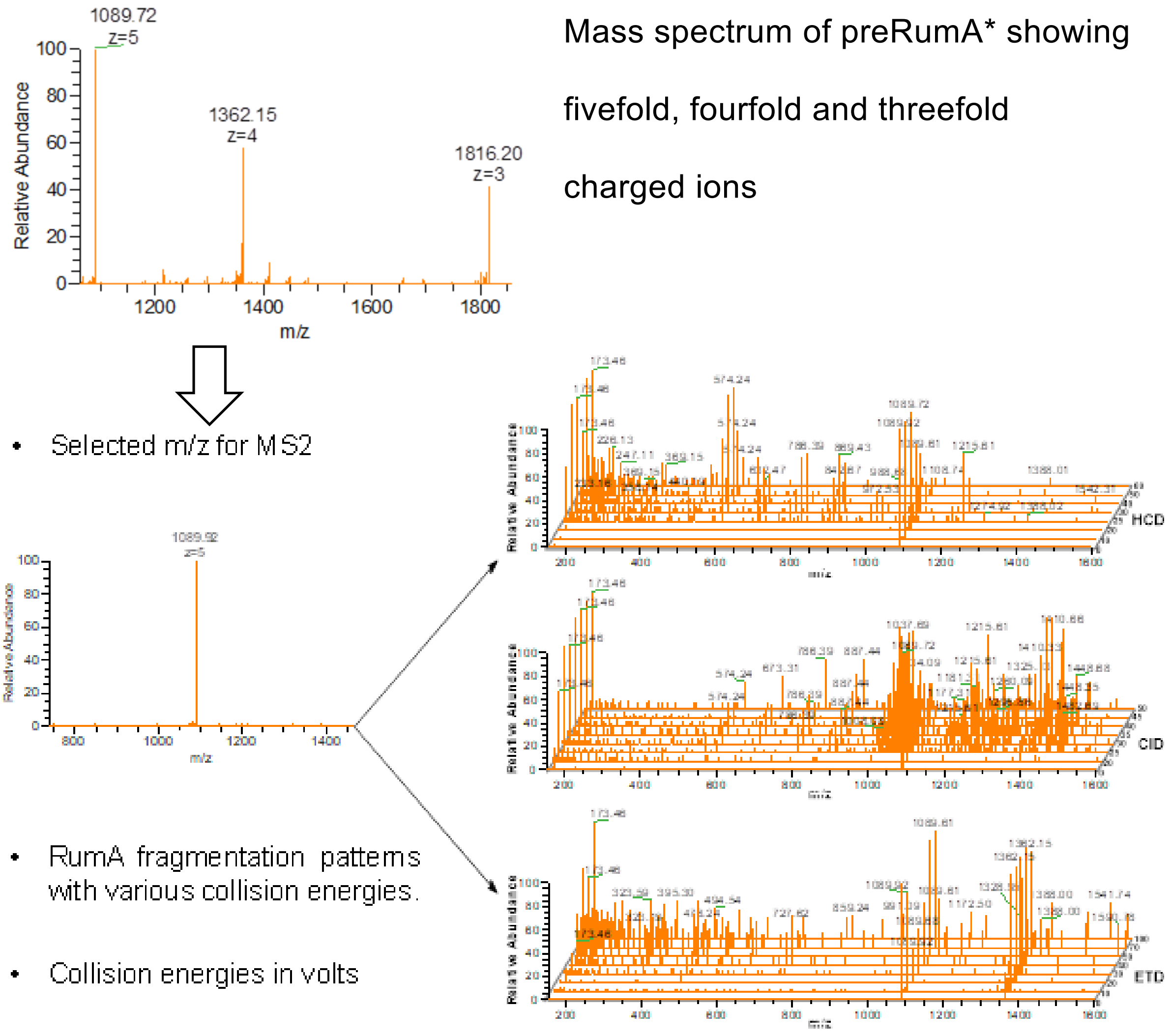

Supplementary Figure 6│Different fragmentation methods and energies used in MS^2 of preRumA*. The most abundant ion was selected for the MS^2 analysis. Fragmentation was energy (in Volts)-dependent and showing that the peptide is resistant to fragmentation especially at low collisional energies. HCD and CID produced more fragmented ions than ETD.

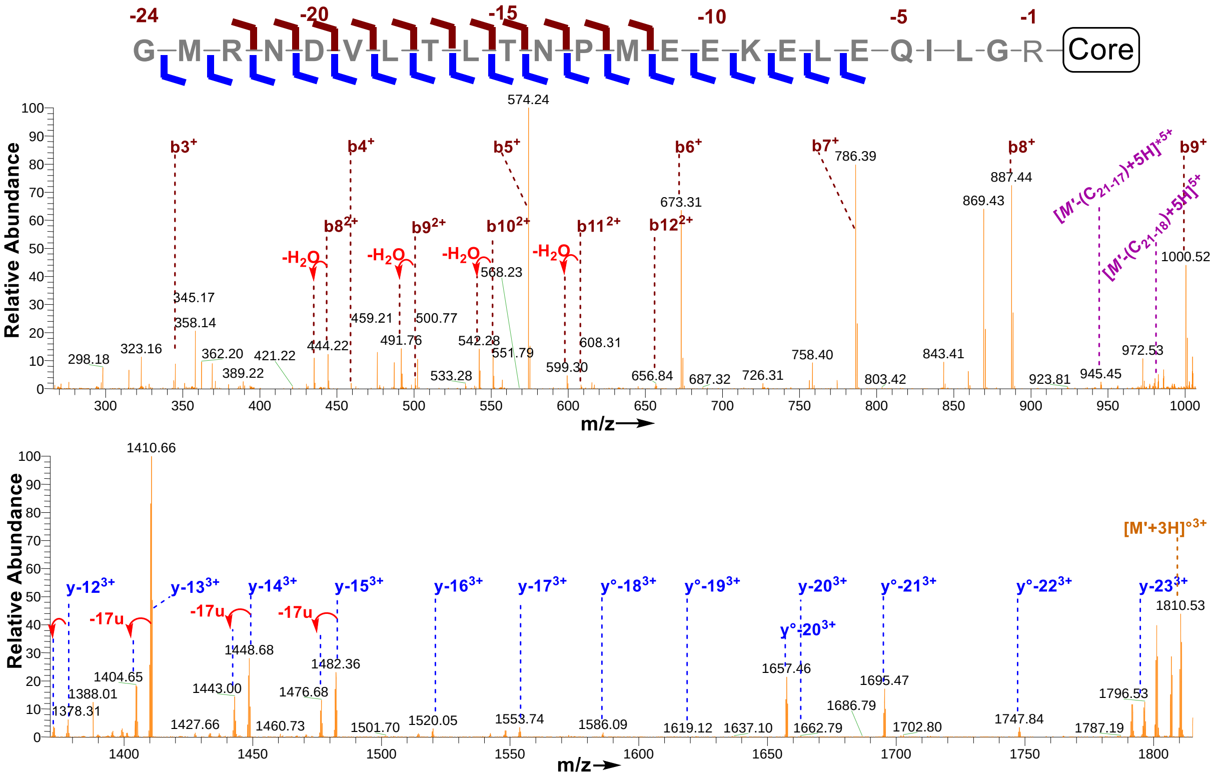

Supplementary Figure 7│Tandem MS^2-experiments and assignment of preRumA* fragment ion peaks. Orbitrap Fusion MS^2 spectra representing ion series with intensive peaks produced from fragmentation of the N-terminal leader peptide of preRumA*. $[M'+5H]^{5+} = 1089.72$.

4. Optimization of His6-RumM and His6-GFP-TEV-preRumA* expression

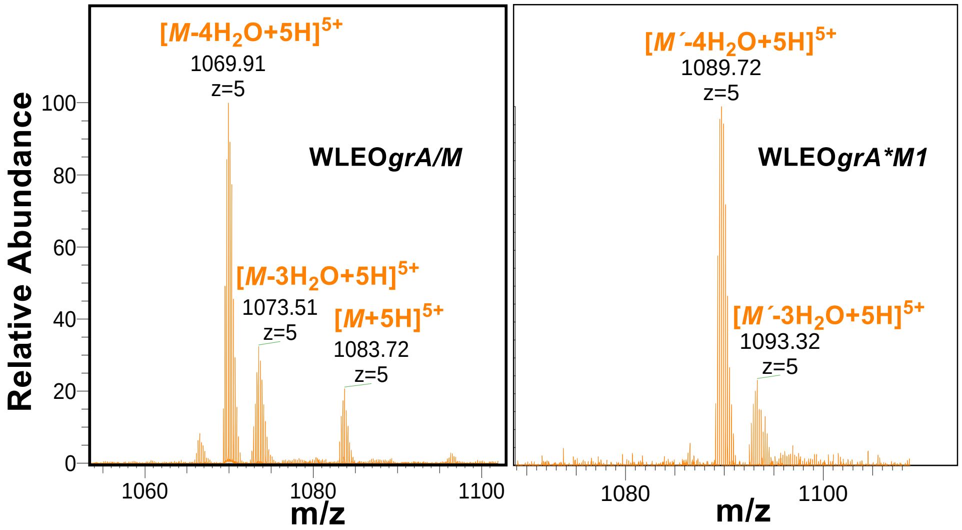

Supplementary Figure 8 | The quality of enhanced preRumA. Comparison between preRumA and preRumA* purified from strain WLEO*grA/M* and strain WLEO*grA*M1*, respectively, with regards to intermediate products formation.

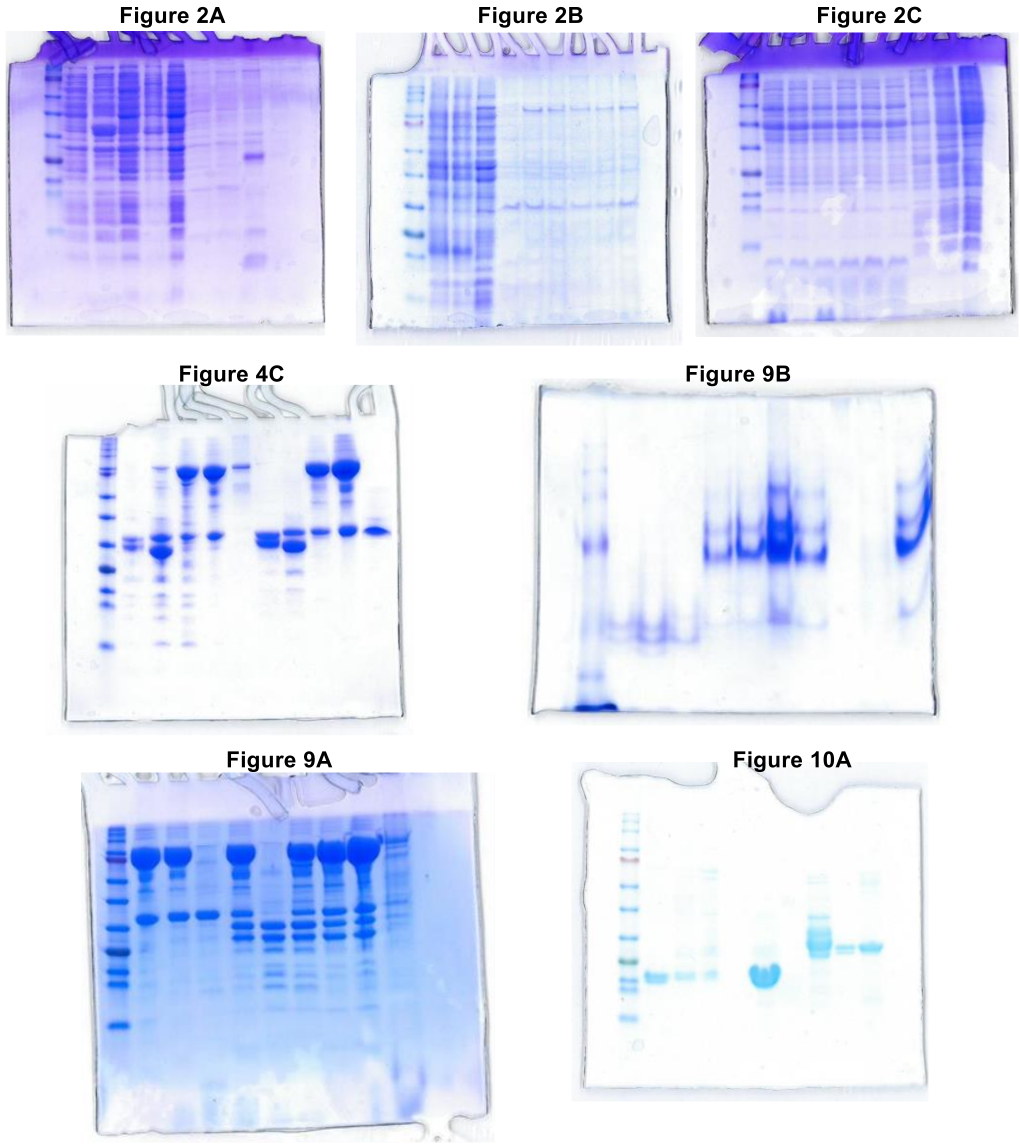

Supplementary Figure 9 | Full scans of gels used main text

References

Agustin, J., Rosenstein, R., Wieland, B., Schneider, U., Schnell, N., Engelke, G., et al. (1992). Genetic analysis of epidermin biosynthetic genes and epidermin-negative mutants of *Staphylococcus epidermidis*. *Eur. J. Biochem.* 204, 1149–1154.

Helfrich, M., Entian, K.-D., and Stein, T. (2007). Structure-function relationships of the lanthionine cyclase SpaC involved in biosynthesis of the *Bacillus subtilis* peptide antibiotic subtilin. *Biochemistry* 46, 3224–3233.

Li, B., and van der Donk, W. A. (2007). Identification of essential catalytic residues of the cyclase NisC involved in the biosynthesis of nisin. *J. Biol. Chem.* 282, 21169–21175.

Li, B., Yu, J. P. J., Brunzelle, J. S., Moll, G. N., Van der Donk, W. A., and Nair, S. K. (2006). Structure and mechanism of the lantibiotic cyclase involved in nisin biosynthesis. *Science (80-.).* 311, 1464–1467.
